# Synthesis, crystal structure and properties of tetra­kis­(pyridine-3-carbo­nitrile)­dithio­cyanatoiron(II) and of diaqua­bis­(pyridine-3-carbo­nitrile)­di­thio­cyanatoiron(II) pyridine-3-carbo­nitrile monosolvate

**DOI:** 10.1107/S205698902300909X

**Published:** 2023-10-31

**Authors:** Christian Näther, Asmus Müller-Meinhard, Inke Jess

**Affiliations:** a Institut für Anorganische Chemie, Universität Kiel, Max-Eyth.-Str. 2, 24118 Kiel, Germany; University of Aberdeen, United Kingdom

**Keywords:** crystal structure, iron thio­cyanate, 3-cyano­pyridine, thermal properties, IR spectrum

## Abstract

The reaction of iron thio­cyanate with 3-cyano­pyridine (C_6_H_4_N_2_) leads to the formation of two compounds with the composition [Fe(NCS)_2_(C_6_H_4_N_2_)_4_] (**1**) and [Fe(NCS)_2_(C_6_H_4_N_2_)_2_(H_2_O)_2_]·2C_6_H_4_N_2_ (**2**). The asymmetric unit of **1** consists of one iron cation, two thio­cyanate anions and four 3-cyano­pyridine ligands in general positions. The iron cation is octa­hedrally coordinated by two N-bonded thio­cyanate anions and four 3-cyano­pyridine ligands. The complexes are arranged in columns along the crystallographic *c*-axis direction and are linked by weak C—H⋯N inter­actions. In **2**, the asymmetric unit consists of one iron cation on a center of inversion as well as one thio­cyanate anion, one 3-cyano­pyridine ligand, one water ligand and one 3-cyano­pyridine solvate mol­ecule in general positions. The iron cation is octa­hedrally coordinated by two N-bonded thio­cyanate anions, two cyano­pyridine ligands and two water ligands. O—H⋯N and C—H⋯S hydrogen bonding is observed between the water ligands and the solvent 3-cyano­pyridine mol­ecules. In the crystal structure, alternating layers of the iron complexes and the solvated 3-cyano­pyridine mol­ecules are observed. Powder X-ray (PXRD) investigations reveal that both compounds were obtained as pure phases and from IR spectroscopic measurements conclusions on the coordination mode of the thio­canate anions and the cyano­group were made. Thermogravimetric (TG) and differential thermoanalysis (DTA) of **1** indicate the formation of a compound with the composition {[Fe(NCS)_2_]_3_(C_6_H_4_N_2_)_4_}_*n*_ that is isotypic to the corresponding Cd compound already reported in the literature. TG/DTA of **2** show several mass losses. The first mass loss corresponds to the removal of the two water ligands leading to the formation of **1**, which transforms into {[Fe(NCS)_2_]_3_(C_6_H_4_N_2_)_4_}_*n*_, upon further heating.

## Abstract 2

Two complexes, **1** and **2**, based on Fe(NCS)_2_ and 3-cyano­pyridine as coligand were synthesized, structurally characterized and investigated for their thermal behavior. In both compounds the Fe^II^ cations are octa­hedrally coordinated by two N-bonded thio­cyanate anions in *trans*-position as well as four 3-cyano­pyridine coligands for compound **1** and two 3-cyano­pyridine ligands and two water mol­ecules for compound **2**. Upon heating, both complexes transform into an inter­mediate with bridging anionic ligands that is isotypic to its Cd(NCS)_2_ and Mn(NCS)_2_ analogs.

## 1. Chemical context

For several years, we and others have been inter­ested in the synthesis, structures and physical properties of coordination compounds based on transition-metal thio­cyanates with additional neutral organic coligands. In such compounds, the anionic ligands can be terminally coordinated to the metal cations or they can act as bridging ligands, leading to the formation of networks (Kabešová & Gažo, 1980 ▸). The latter compounds are of special inter­est because different magnetic phenomena can be observed (González *et al.*, 2012 ▸; Werner *et al.*, 2014 ▸; Palion-Gazda *et al.*, 2015 ▸; Mautner *et al.*, 2018 ▸; Rams *et al.*, 2020 ▸). Unfortunately, the compounds with a bridging coordination are sometimes difficult to prepare with metal cations such as Mn, Fe, Co or Ni, because these cations are less chalcophilic, which means that a terminal coordination is preferred. In such cases, an alternative synthetic approach can be used based on thermal treatment of suitable precursor compounds, which we developed many years ago for the synthesis of copper(I) halide coordination polymers (Näther *et al.*, 2001 ▸; Näther & Jess, 2004 ▸). For the synthesis of thio­cyanate coordination polymers, these precursors consist of compounds in which the metal cations are octa­hedrally coordinated by two terminally N-bonding thio­cyanate anions and four coligands that in most cases consist of pyridine derivatives. If such compounds are heated, the coligands are frequently stepwise removed and the empty coordination sites at the metal centers are completed by the S atoms of the anionic ligands that in the complex do not participate in the metal coordination, which enforces a bridging coordination of the thio­cyanate anions. Major advantages of this approach are the fact that this reaction is irreversible, that the products are formed in qu­an­ti­tative yields, and that in several cases, polymorphic or isomeric modifications can be prepared (Werner *et al.*, 2015 ▸). However, following this approach, only microcrystalline powders are observed that cannot be investigated by single crystal X-ray diffraction. In this case, the corresponding Cd(NCS)_2_ compounds can be prepared, which also prefer an octa­hedral coordination. Because cadmium is more chalcophilic than the cations mentioned above, the synthesis of compounds with a bridging coordination is easier and, in most cases, they can easily be crystallized and characterized by single-crystal structure analysis (Wöhlert *et al.*, 2013 ▸). In several cases they are isotypic with the Mn, Fe, Co or Ni compounds, allowing the structural identification of the latter. Moreover, with Cd(NCS)_2_ and one definite ligand, usually several compounds with a different, in part unusual ratio between Cd(NCS)_2_ and the coligands can be obtained. If such compounds are detected, one can determine whether they are also available with other metal cations.

In this context, we have reported new thio­cyanate coord­in­ation compounds based on Cd(NCS)_2_ and 3-cyano­pyridine as ligand, where five different compounds were detected (Jochim *et al.*, 2020 ▸). This includes two solvates with the composition [Cd(NCS)_2_(C_6_H_4_N_2_)_2_]_*n*_·C_6_H_4_N_2_ and [Cd(NCS)_2_(C_6_H_4_N_2_)_2_]_*n*_·1/3C_6_H_4_N_2_ (C_6_H_4_N_2_ = 3-cyano­pyridine) and one further compound with a similar structure with the composition [Cd(NCS)_2_(C_6_H_4_N_2_)_2_]_*n*_. In all of these compounds, the Cd cations are octa­hedrally coordinated by two thio­cyanate anions and four 3-cyano­pyridine coligands and are linked by pairs of μ-1,3-bridging thio­cyanate anions into chains, which is a common motif in thio­cyanate coordination polymers. Two additional 3-cyano­pyridine deficient compounds with an unusual ratio between Cd(NCS)_2_ and 3-cyano­pyridine were also characterized. In {[Cd(NCS)_2_]_2_(C_6_H_4_N_2_)_3_}_*n*_ and {[Cd(NCS)_2_]_3_(C_6_H_4_N_2_)_4_}_*n*_ the cations are also octa­hedrally coordinated and linked into chains, but some of the 3-cyano­pyridine ligands act as bridging ligands and connect the chains into layers.

In further work, corresponding compounds with Ni(NCS)_2_ were investigated. With this cation, discrete complexes with the composition Ni(NCS)_2_(C_6_H_4_N_2_)_4_ have already been reported in the literature (Kilkenny & Nassimbeni, 2001 ▸), Ni(NCS)_2_(C_6_H_4_N_2_)_2_(H_2_O)_2_, Ni(NCS)_2_(C_6_H_4_N_2_)_2_(CH_3_OH)_2_ and Ni(NCS)_2_(C_6_H_4_N_2_)_2_(CH_3_CN)_2_ were prepared in which the metal cations are always octa­hedrally coordinated (Krebs *et al.*, 2021 ▸). All of these complexes transform into a new compound with the composition Ni(NCS)_2_(C_6_H_4_N_2_)_2_ upon heating, which can also be prepared from solution. In this compound, the metal cations are linked by pairs of μ-1,3-bridging thio­cyanate anions into dinuclear units that are further connected by single anionic ligands into layers. Therefore, the structures of the Ni(NCS)_2_ compounds are completely different from those of the Cd(NCS)_2_ compounds.

Compounds with Mn(NCS)_2_ and 3-cyano­pyridine were prepared because Mn^II^ compounds frequently behave similar to Cd(NCS)_2_ compounds (Krebs *et al.*, 2023 ▸). With Mn(NCS)_2_ compounds with the composition Mn(NCS)_2_(C_6_H_4_N_2_)_4_, Mn(NCS)_2_(C_6_H_4_N_2_)_2_(H_2_O)_2_·bis­(C_6_H_4_N_2_) solvate and Mn(NCS)_2_(C_6_H_4_N_2_)(H_2_O) and Mn(NCS)_2_(C_6_H_4_N_2_)_2_(H_2_O)_2_ were obtained, but the latter compound cannot be prepared as a pure phase. Most compounds consist of discrete complexes but in Mn(NCS)_2_(C_6_H_4_N_2_)(H_2_O) the Mn cations are linked by single μ-1,3-bridging thio­cyanates into chains, which are further connected into layers by the 3-cyano­pyridine coligands. Thermoanaytical investigations reveal that the discrete complex Mn(NCS)_2_(C_6_H_4_N_2_)_4_ transforms into a new compound with the composition [(Mn(NCS)_2_)_3_(C_6_H_4_N_2_)_4_]_*n*_ that is isotypic to the corresponding Cd compound mentioned above. When Mn(NCS)_2_(C_6_H_4_N_2_)_2_(H_2_O)_2_·bis­(C_6_H_4_N_2_) solvate is heated, it transforms into [(Mn(NCS)_2_)_3_(C_6_H_4_N_2_)_4_]*_n_ via* the discrete complex Mn(NCS)_2_(C_6_H_4_N_2_)_4_ as an inter­mediate. Therefore, the structural behavior and the thermal reactivity is much more similar to that of the Cd(NCS)_2_ compounds with 3-cyano­pyridine as coligand.

Based on all these findings, we decided to prepare corresponding compounds based on Fe(NCS)_2_ and 3-cyano­pyridine to investigate if this cation behaves more similarly to Cd^II^, Mn^II^ or Ni^II^. Within this systematic work, only two discrete complexes were obtained, which were investigated for their thermal behavior. 
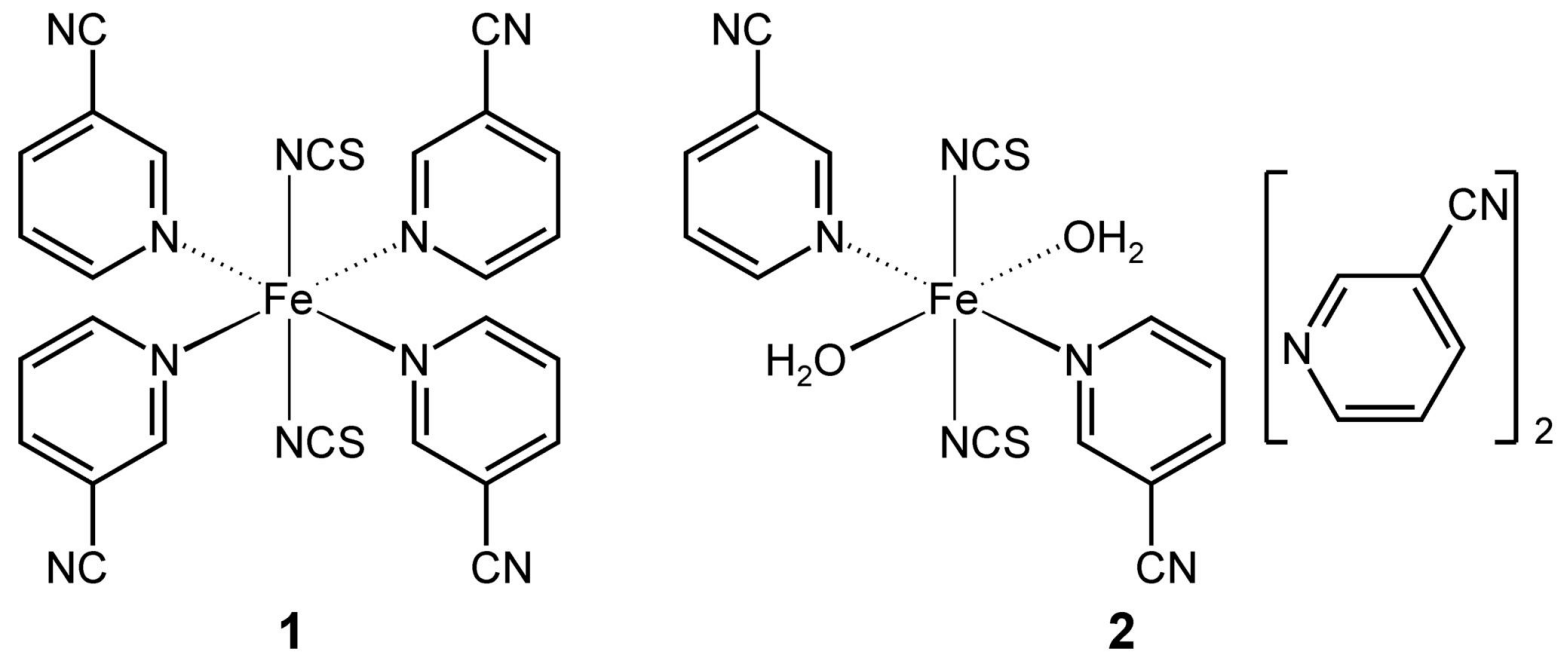


## 2. Structural commentary

**Figure 1:**
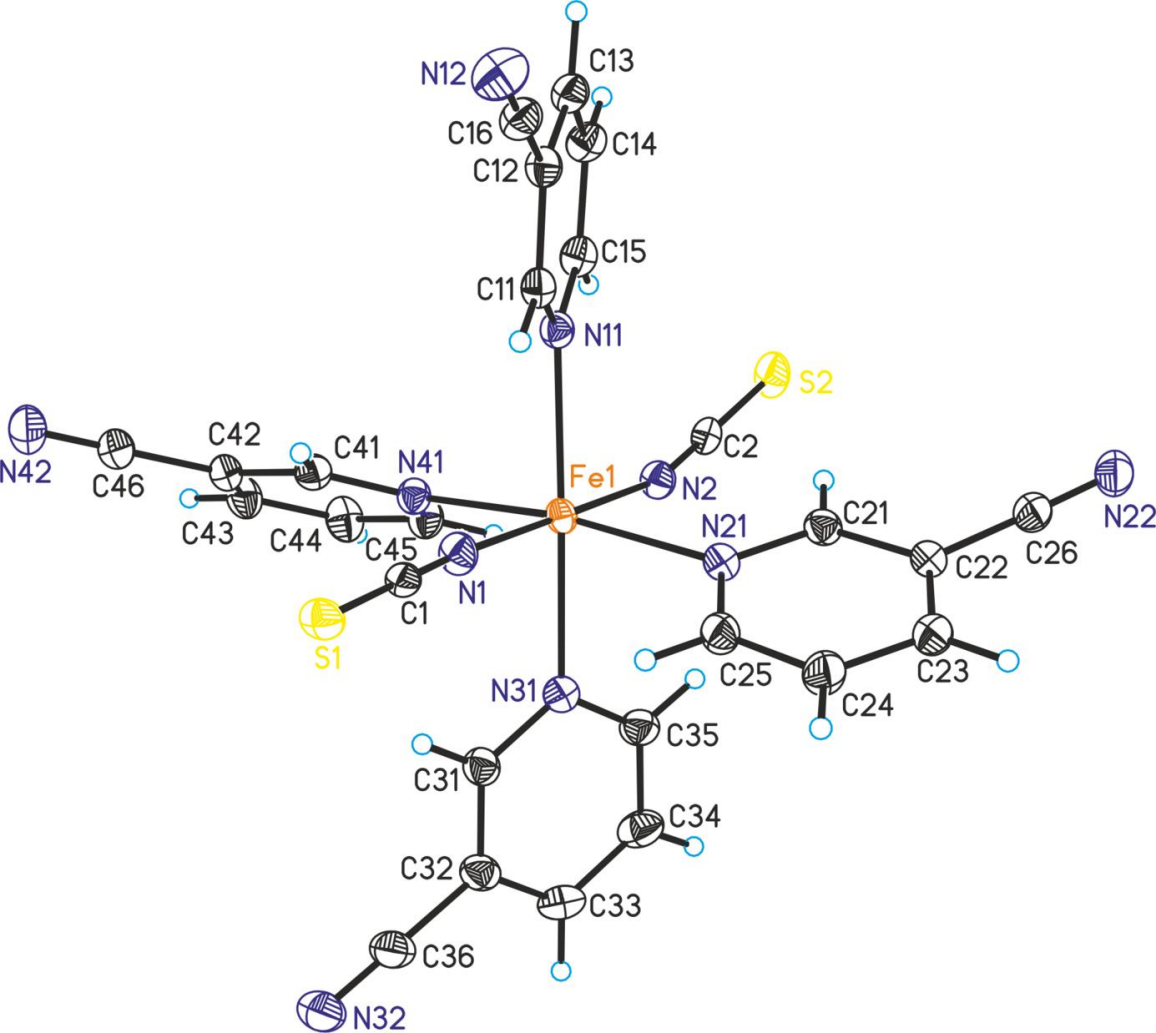
The mol­ecular structure of **1** with displacement ellipsoids drawn at the 50% probability level.

The asymmetric unit of Fe(NCS)_2_(C_6_H_4_N_2_)_4_ (**1**) consists of one iron cation as well as of two thio­cyanate anions and four 3-cyano­pyridine coligands in general positions (Fig. 1 ▸). The iron cations are octa­hedrally coordinated by two terminally N-bonded thio­cyanate anions and four 3-cyano­pyridine colig­ands that coordinate *via* the pyridine N atom to the metal centers (Fig. 1 ▸). This compound is isotypic to Ni(NCS)_2_(C_6_H_4_N_2_)_4_, Mn(NCS)_2_(C_6_H_4_N_2_)_4_ and Zn(NCS)_2_(C_6_H_4_N_2_)_4_ already reported in the literature (Kilkenny & Nassimbeni, 2001 ▸; Krebs *et al.*, 2021 ▸, Krebs *et al.*, 2023 ▸; Jochim *et al.*, 2019 ▸). Despite differences because of the different ionic radii, the bond lengths are comparable to those in the isotypic compounds (Table 1 ▸). From the N—Fe—N bond angles it is obvious that the octa­hedra are slightly distorted (Table 1 ▸).

**Figure 2:**
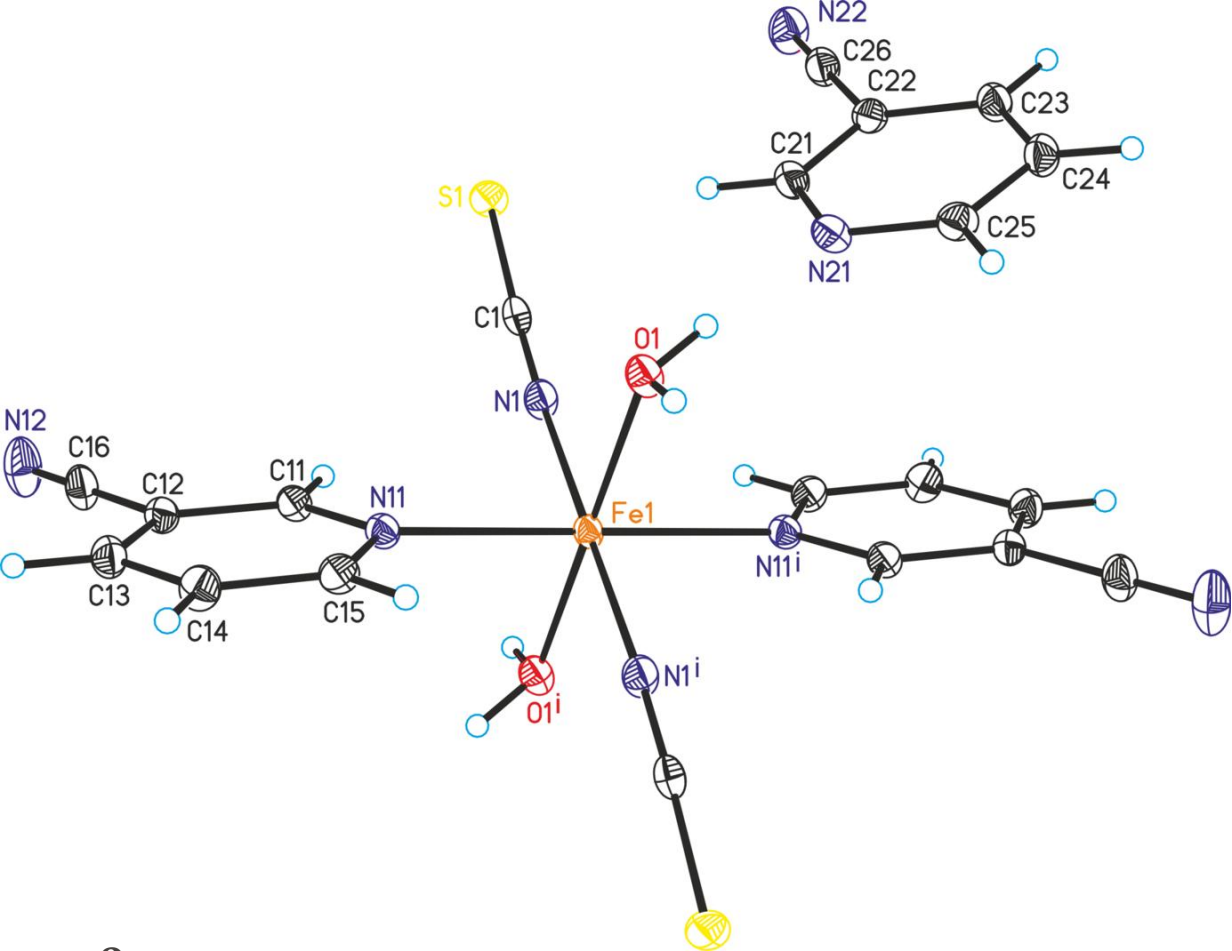
The mol­ecular structure of **2** with displacement ellipsoids drawn at the 50% probability level. Symmetry codes for the generation of equivalent atoms: (i) −*x* + 1, −*y* + 1, −*z* + 1.

In Fe(NCS)_2_(C_6_H_4_N_2_)_2_(H_2_O)_2_·2(C_6_H_4_N_2_) (**2**), the asymmetric unit consists of one iron cation that is located on a center of inversion as well as one thio­cyanate anion, one 3-cyano­pyridine ligand, one water ligand and one 3-cyano­pyridine solvate mol­ecule in general positions (Fig. 2 ▸). The iron cation is octa­hedrally coordinated by two 3-cyano­pyridine coligands that are connected *via* the pyridine N atom to the Fe^II^ cations, two water ligands and two terminally N-bonded thio­cyanate anions. This compound is isotypic to Mn(NCS)_2_(C_6_H_4_N_2_)_2_(H_2_O)_2_·2(C_6_H_4_N_2_) and Zn(NCS)_2_(C_6_H_4_N_2_)_2_(H_2_O)_2_ ·2(C_6_H_4_N_2_) that are reported in the literature (Krebs *et al.*, 2023 ▸; Jochim *et al.*, 2019 ▸). The Fe—*X* (*X* = N, O) bond lengths are slightly shorter than those in the corresponding Mn compound and the bond angles show that the octa­hedra are slightly distorted (Table 2 ▸).

## 3. Supra­molecular features

**Figure 3:**
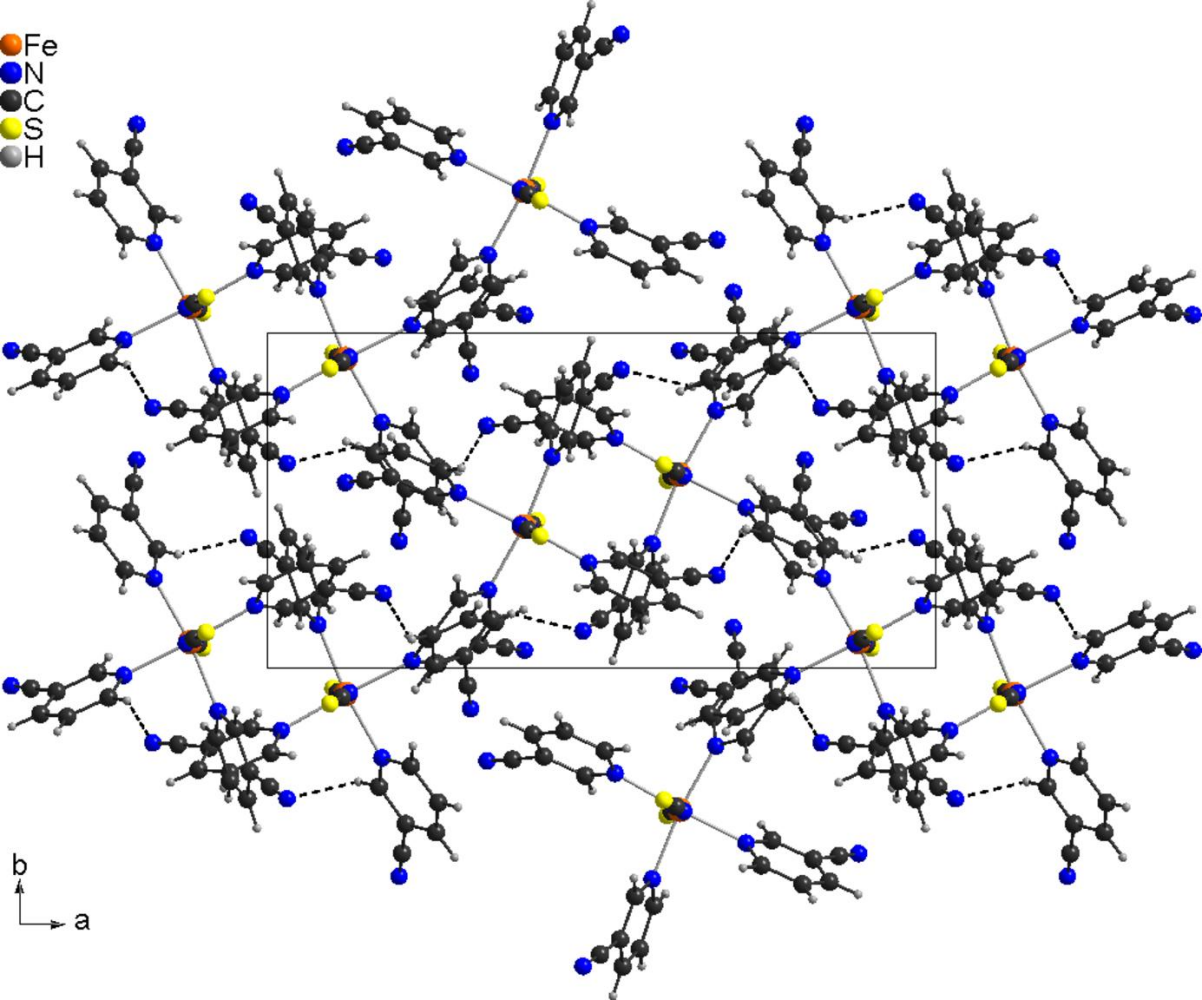
Crystal structure of **1** viewed along the crystallographic *c*-axis direction with C—H⋯N bonds shown as dashed lines.

In compound **1** the discrete complexes are arranged in columns that are oriented along the crystallographic *c*-axis direction (Fig. 3 ▸). Within the columns, neighboring 3-cyano­pyridine rings are not coplanar, with no indication of π–π stacking inter­actions. The complexes are connected *via* weak C—H⋯N hydrogen bonding but most of these inter­actions exhibit C—H⋯N angles far from linearity, indicating that they do not represent strong inter­actions (Table 3 ▸ and Fig. 3 ▸)

**Figure 4:**
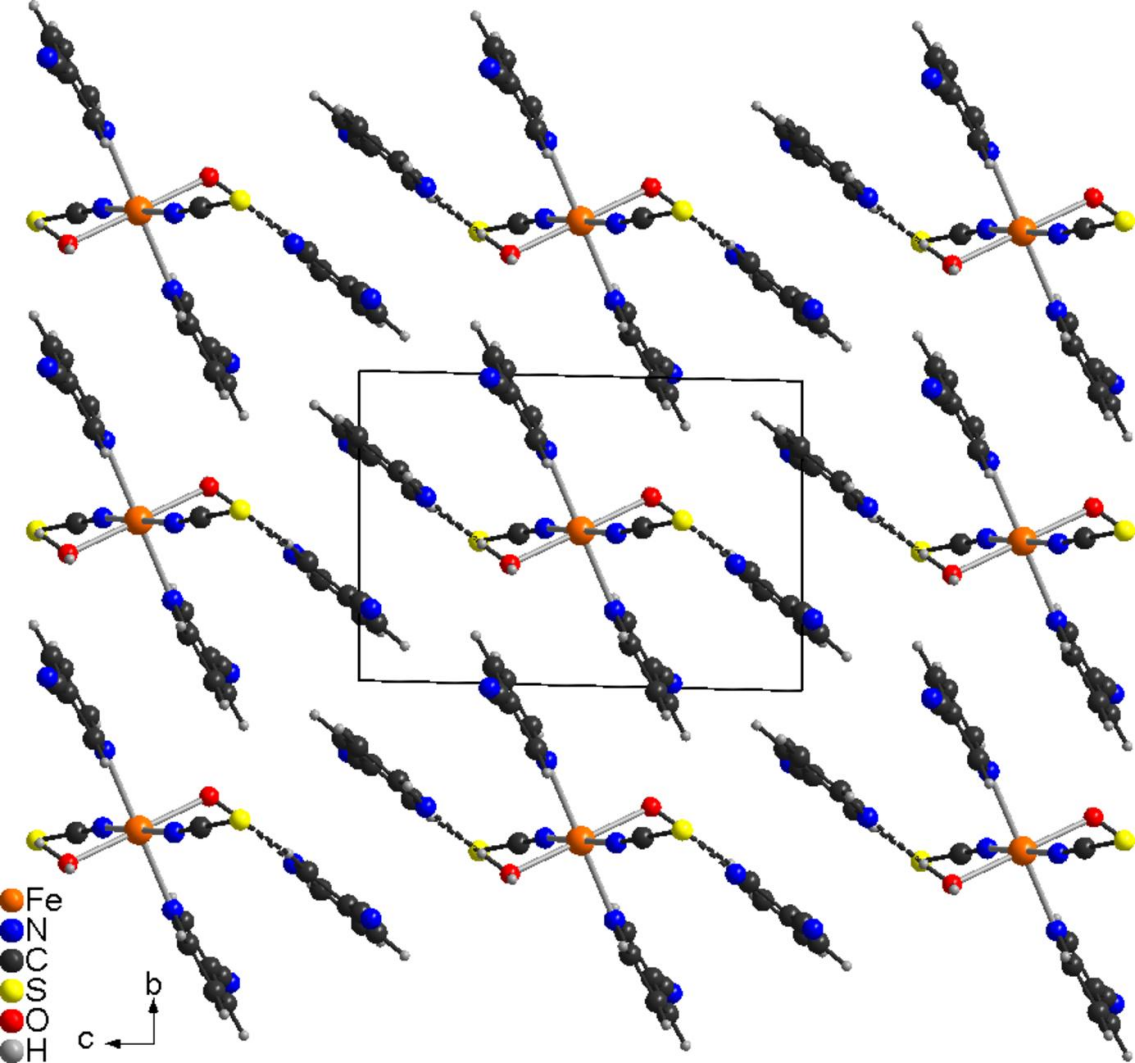
Crystal structure of **2** viewed along the crystallographic *a*-axis direction with C—H⋯S and O—H⋯N hydrogen bonds shown as dashed lines.

In compound **2** the discrete complexes are also stacked in columns that proceed along the crystallographic *a*-axis (Fig. 4 ▸). These columns are arranged in layers that are parallel to the *ab*-plane. The 3-cyano­pyridine solvate mol­ecules are located between these layers and are connected to the complexes *via* C—H⋯S and O—H⋯N hydrogen bonding where the pyridine N atom is involved (Table 4 ▸ and Fig. 4 ▸). There are additional C—H⋯N inter­actions, but from the distances and angles it is obvious that they correspond to only very weak inter­actions. Within the 3-cyano­pyridine layers, neighboring 3-cyano­pyridine mol­ecules are oriented parallel but shifted relative to each other, preventing π–π inter­actions (Fig. 4 ▸).

## 4. Database survey

A search in the CSD (version 5.43, last update November 2023; Groom *et al.*, 2016 ▸) using *ConQuest* (Bruno *et al.*, 2002 ▸) reveals that a number of thio­cyanate coordination compounds with 3-cyano­pyridine have already been reported in the literature and most of these compounds have already been mentioned in the *Chemical context* section above. This includes discrete complexes with the composition *M*(NCS)_2_(C_6_H_4_N_2_)_4_ (*M* = Ni, Zn) in which the metal cations are octa­hedrally coordinated by two thio­cyanate anions and four 3-cyano­pyridine coligands (CSD refcode UDABAC, Kilkenny & Nassimbeni, 2001 ▸; UDABAC01, Krebs *et al.*, 2021 ▸; LIPZES, Jochim *et al.*, 2019 ▸). There are additional complexes with the composition *M*(NCS)_2_(C_6_H_4_N_2_)_4_ (*M* = Ni, Co) that contain solvate mol­ecules (UDABIK, Kilkenny & Nassimbeni, 2001 ▸; UDABEG, Kilkenny & Nassimbeni, 2001 ▸; OBONOK, Diehr *et al.*, 2011 ▸) as well as one complex of composition Zn(NCS)_2_(C_6_H_4_N_2_)_2_(H_2_O)_2_ that also contains solvate mol­ecules (LIZNOA; Jochim *et al.*, 2019 ▸).

Additionally, complexes with the composition Ni(NCS)_2_(C_6_H_4_N_2_)_2_(*X*)_2_ (*X* = MeCN, OCH_3_, H_2_O, OHCH_3_) are reported, in which the nickel cations are octa­hedrally coordinated by two thio­cyanate anions, two 3-cyano­pyridine coligands and two further coligands (YAXDOU, Krebs *et al.*, 2021 ▸; YAXDIO, Krebs *et al.*, 2021 ▸; YAXCUZ, Krebs *et al.*, 2021 ▸). With Cu^II^, an aqua complex with the composition Cu(NCS)_2_(C_6_H_4_N_2_)_2_(H_2_O)_2_ is also found (ABOVAR; Handy *et al.*, 2017 ▸). One complex of the composition Zn(NCS)_2_(C_6_H_4_N_2_)_2_ is reported in which the zinc cations are tetra­hedrally coordinated by two thio­cyanate anions and two 3-cyano­pyridine coligands (LIZNUG; Jochim *et al.*, 2019 ▸).

Furthermore, one structure of the composition Ni(NCS)_2_(C_6_H_4_N_2_)_2_ exists in which nickel cations are octa­hedrally coordinated by four thio­cyanate anions and two 3-cyano­pyridine coligands. The nickel cations are linked by pairs of thio­cyanate anions into dinuclear units that are further connected into layers by single bridging anionic ligands (YAXDEK; Krebs *et al.*, 2021 ▸). In a further compound of the composition Cd(NCS)_2_(C_6_H_4_N_2_)_2_, the cadmium cations are octa­hedrally coordinated by four thio­cyanate anions and two 3-cyano­pyridine coligands and are linked through two thio­cyanate anions into chains (NURTUS; Jochim *et al.*, 2020 ▸). Two additional compounds with similar chain structures are also listed that contain 3-cyano­pyridine solvate mol­ecules (NURTOM, Jochim *et al.*, 2020 ▸; NURTIG, Jochim *et al.*, 2020 ▸). With Cd(NCS)_2_, two additional compounds are reported in which Cd(NCS)_2_ chains are linked by some of the 3-cyano­pyridine ligands into layers (NURVAA and NURVEE; Jochim *et al.*, 2020 ▸). With Mn(NCS)_2_, the previously mentioned compounds with the composition Mn(NCS)_2_(C_6_H_4_N_2_)_4_, Mn(NCS)_2_(C_6_H_4_N_2_)_2_(H_2_O)_2_-bis­(C_6_H_4_N_2_) solvate and Mn(NCS)_2_(C_6_H_4_N_2_)(H_2_O) and Mn(NCS)_2_(C_6_H_4_N_2_)_2_(H_2_O)_2_ have also been reported (Krebs *et al.*, 2023 ▸) but these are not yet listed in the CSD.

## 5. Physical characterization investigations

**Figure 5:**
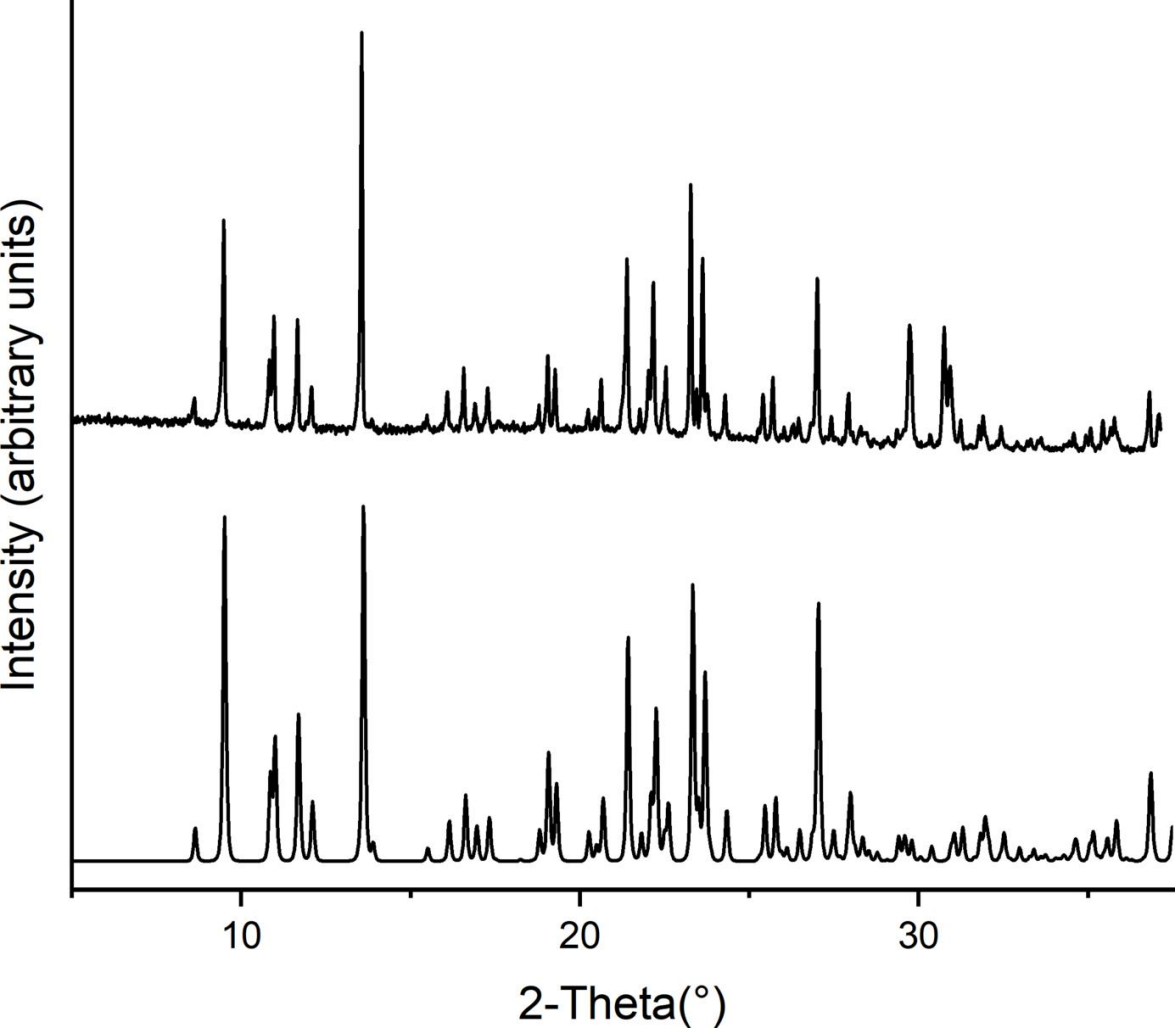
Experimental (top) and calculated PXRD patterns (bottom) of **1**.

Comparison of the experimental powder pattern of **1** and **2** with that calculated from single crystal data shows that both compounds were obtained as pure phases (Figs. 5 ▸ and 6 ▸). For compound **1**, the CN stretching vibration of the thio­cyanate anion is observed at 2056 cm^−1^ and for the cyano­group of the 3-cyano­pyridine ligand at 2234 cm^−1^ while for compound **2** these values amount to 2238 cm^−1^ and 2080 cm^−1^, which is in agreement with the fact that the thio­cyanate anions are only terminally coordinated and that the cyano­group is not involved in the metal coordination (Figs. S1 and S2).

**Figure 7:**
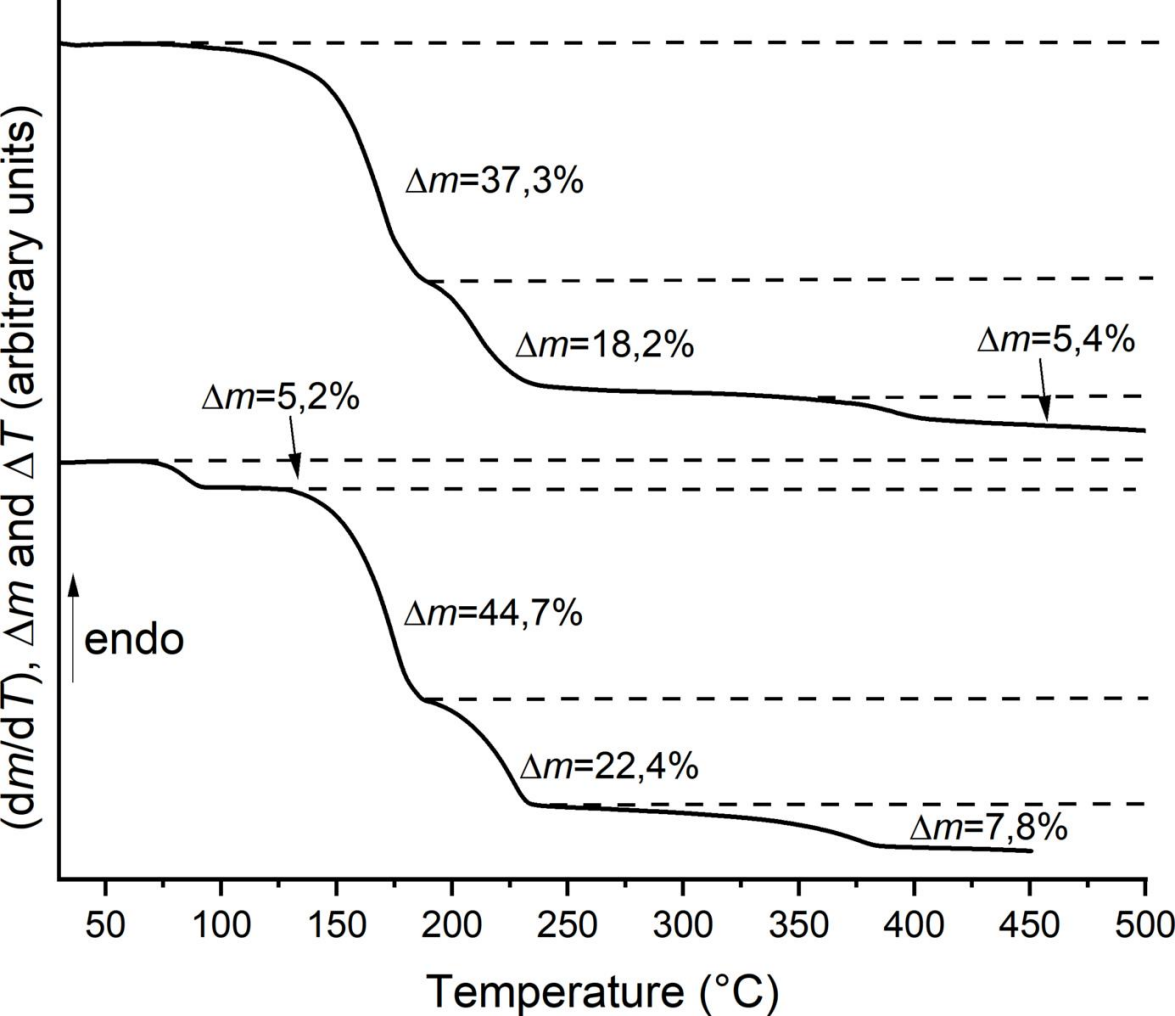
TG curves for **1** (top) and **2** (bottom) measured with a 4 °C min^−1^ heating rate. The mass losses are stated in %.

The thermal properties of both compounds were investigated by simultaneous thermogravimetry and differential thermoanalysis (TG–DTA). For compound **1** the measurements reveal three mass losses due to heating that are accompanied with two endothermic (first and second mass loss) and one exothermic (third mass loss) events in the DTA curve (Fig. 7 ▸ and S3). From the first derivative of the TG curve it is obvious that all mass losses are not well resolved. The first mass loss of 37.3% is slightly higher that that calculated for the removal of two 3-cano­pyridine ligands (Δ*m*_calc._= 35.4%). To identify the inter­mediate formed after the first mass loss we repeated the TG measurement and isolated the residue after the respective mass loss. The residue was then investigated by IR spectroscopy and powder X-ray diffraction (PXRD). The CN stretching vibrations of the thio­cyanate anions are observed at 2105 cm^−1^ and at 2078cm^−1^, which indicates that μ-1,3-bridging anionic ligands are present (Fig. S4). For the cyano group, two different values at 2248 cm^−1^ and 2270 cm^−1^ are observed, indicating that some of them are coordinated to the metal center, whereas some others are not (Fig. S4). If the experimental powder pattern is compared with those calculated for all thio­cyanate compounds with less 3-cyano­pyridine (Fig. S5) that are reported in the literature (see *Database survey*), it is evident that this crystalline phase is isotypic to compounds {[Cd(NCS)_2_]_3_(C_6_H_4_N_2_)_4_}_*n*_ (Jochim *et al.*, 2020 ▸) and {[Mn(NCS)_2_]_3_(C_6_H_4_N_2_)_4_}_*n*_ (Krebs *et al.*, 2023 ▸) already reported in the literature (Fig. S5). In this context, it is surprising that two different CN stretching vibrations for the thio­cyanate anions are observed, because this structure contains only one crystallographically independent anion, but similar observations were made for the corresponding Mn compound (Krebs *et al.*, 2023 ▸). However, in the second mass loss the remaining 3-cyano­pyridine ligands are removed and upon further heating Mn(NCS)_2_ decomposes.

For compound **2**, four mass losses were observed upon heating that are accompanied with three endothermic and one exothermic events in the DTA curve (Figs. 7 ▸ and S6). The first mass loss of 5.2% is in good agreement with the loss of two water ligands (Δ*m*_calc._= 5.8%). This indicates that compound **1** has been formed. To prove this assumption, a second TG measurement was performed in which the residue formed after the first mass loss was isolated and investigated by IR spectroscopy and PXRD. The IR spectra is very similar to that of compound **1** (compare Figs. S1 and S7) and comparison of the experimental pattern with that calculated for **1** proves that this compound was obtained (Fig. S8). The second mass loss of 44.7% is in excellent agreement with the loss of 2.67 3-cyano­pyridine ligands (Δ*m*_calc._= 44.5%), which indicates that after the second mass loss {[Fe(NCS)_2_]_3_(C_6_H_4_N_2_)_4_}_*n*_ has been formed. This assumption has been proved through a repetition of the TG measurement, isolation of the residue after the second mass loss and by IR (Fig. S9) as well as PXRD investigations (Fig. S10).

## 6. Synthesis and crystallization

FeSO_4_·7H_2_O and KSCN were purchased from Sigma-Aldrich and 3-cyano­pyrine was purchased from Alfa Aesar.

A microcrystalline powder of **1** was obtained by the reaction of 0.25 mmol of FeSO_4_·7 H_2_O (69.5 mg), 0.5 mmol of KSCN (48.6 mg) and 1 mmol (104.1 mg) of 3-cyano­pyridine in 0.5 ml of ethanol. The mixture was stirred for 1 d at room temperature and filtered off. Crystals suitable for single crystal X-ray diffraction were obtained with the same amount of reactants and solvent under hydro­thermal conditions (400 K for 1 d) without stirring.

For **2**, a microcrystalline powder was obtained by the reaction of 1 mmol of FeSO_4_·7H_2_O (278 mg), 2 mmol of KSCN (194 mg) and 2 mmol (208.2 mg) of 3-cyano­pyridine in 1.5 ml of water. The mixture was filtered off after stirring at room temperature for 2 d. To obtain crystals for singe-crystal X-ray diffraction, 0.25 mmol of FeSO_4_·7H_2_O (69.5 mg), 0.5 mmol of KSCN (48.6 mg) and 1 mmol (104.1 mg) of 3-cyano­pyridine were mixed in 1.5 ml of water and heated for 2 d at 403 K under hydro­thermal conditions.

IR spectra of **1** and **2** can be found in Figs. S1 and S2.

## 7. Refinement

Crystal data, data collection and structure refinement details are summarized in Table 5 ▸. The C-bound H atoms were positioned with idealized geometry and were refined isotropically with *U*_ĩso_(H) = 1.2*U*_eq_(C) using a riding model. The water H atoms were located in a difference map and refined isotropically with freely varying coordinates.

## Supplementary Material

Crystal structure: contains datablock(s) 1, 2. DOI: 10.1107/S205698902300909X/hb8079sup1.cif

Structure factors: contains datablock(s) 1. DOI: 10.1107/S205698902300909X/hb80791sup2.hkl

Structure factors: contains datablock(s) 2. DOI: 10.1107/S205698902300909X/hb80792sup3.hkl

IR spectrum of compound 1. Given is the value of the CN stretching vibration of the thiocyanate anions and the cyanogroup of the 3-cyanopyridine ligand. DOI: 10.1107/S205698902300909X/hb8079sup4.png

IR spectrum of compound 2. Given is the value of the CN stretching vibration of the thiocyanate anions and the cyanogroup of the 3-cyanopyridine ligand. DOI: 10.1107/S205698902300909X/hb8079sup5.png

DTG, TG and DTA curve of 1. DOI: 10.1107/S205698902300909X/hb8079sup6.png

IR spectrum of the residue obtained after the first mass loss in a TG measurement of 1. Given are the values of the CN stretching vibration of the thiocyanate anions and the cyanogroup of the 3-cyanopyridine ligand. DOI: 10.1107/S205698902300909X/hb8079sup7.png

Experimental powder pattern of the residue obtained after the first mass loss in a TG measurement of 1 (top) and calculated pattern for {[Cd(NCS)2]3(3-cyanopyridine)4}n. DOI: 10.1107/S205698902300909X/hb8079sup8.png

DTG, TG and DTA curve of 2. DOI: 10.1107/S205698902300909X/hb8079sup9.png

IR spectrum of the residue obtained after the first mass loss in a TG measurement of 2. Given is the value of the CN stretching vibration of the thiocyanate anions and the cyanogroup of the 3-cyanopyridine ligand. DOI: 10.1107/S205698902300909X/hb8079sup10.png

Experimental powder pattern of the residue obtained after the first mass loss in a TG measurement of 2 (top) and calculated pattern for 1. DOI: 10.1107/S205698902300909X/hb8079sup11.png

IR spectrum of the residue obtained after the second mass loss in a TG measurement of 2. Given are the values of the CN stretching vibration of the thiocyanate anions and the cyanogroup of the 3-cyanopyridine ligand. DOI: 10.1107/S205698902300909X/hb8079sup12.png

Experimental powder pattern of the residue obtained after the second mass loss in a TG measurement of 2 (top) and calculated pattern for {[Cd(NCS)2]3(3-cyanopyridine)4}n. DOI: 10.1107/S205698902300909X/hb8079sup13.png

CCDC references: 2301450, 2301449

Additional supporting information:  crystallographic information; 3D view; checkCIF report

## Figures and Tables

**Figure 6:**
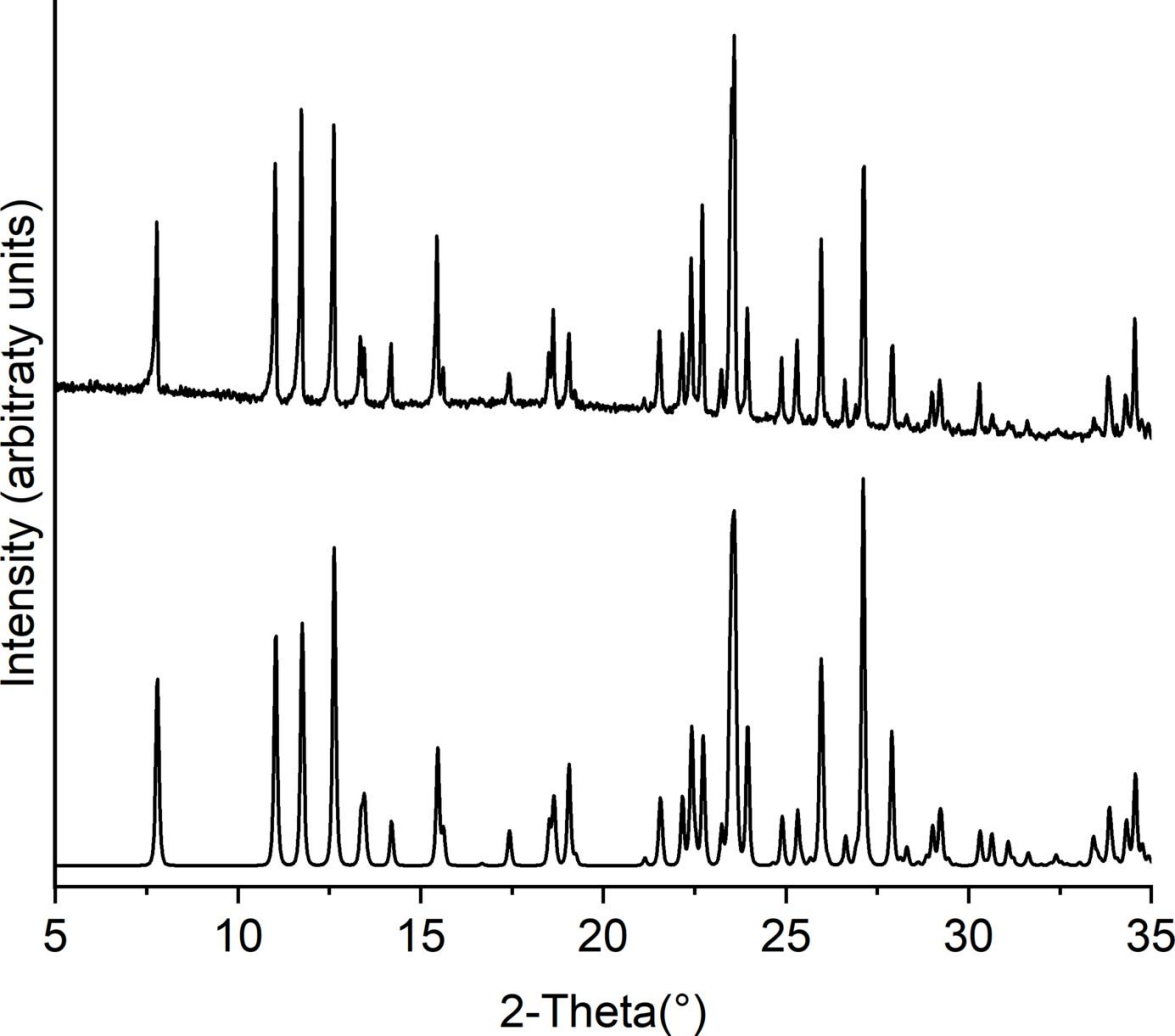
Experimental (top) and calculated PXRD patterns (bottom) of **2**.

**Table 1:** Selected geometric parameters (Å, °) for **1** [Chem scheme1]

Fe1—N1	2.065 (2)	Fe1—N21	2.273 (2)
Fe1—N2	2.069 (2)	Fe1—N31	2.257 (2)
Fe1—N11	2.2660 (19)	Fe1—N41	2.2339 (19)
			
N1—Fe1—N2	179.21 (9)	N11—Fe1—N21	97.44 (7)
N1—Fe1—N11	90.70 (8)	N31—Fe1—N11	176.72 (7)
N1—Fe1—N21	90.56 (8)	N31—Fe1—N21	85.31 (7)
N1—Fe1—N31	91.05 (8)	N41—Fe1—N11	90.12 (7)
N1—Fe1—N41	90.15 (9)	N41—Fe1—N21	172.40 (7)
N2—Fe1—N11	88.70 (8)	N41—Fe1—N31	87.11 (7)
N2—Fe1—N21	89.00 (8)	Fe1—N1—C1	175.0 (2)
N2—Fe1—N31	89.57 (8)	Fe1—N2—C2	163.2 (2)
N2—Fe1—N41	90.37 (8)		

**Table 2:** Selected geometric parameters (Å, °) for **2** [Chem scheme1]

Fe1—N1	2.1207 (10)	Fe1—N11	2.2358 (10)
Fe1—O1	2.1267 (9)		
			
N1—Fe1—O1	89.42 (4)	O1^i^—Fe1—N11	87.62 (3)
N1^i^—Fe1—O1	90.58 (4)	O1—Fe1—N11	92.38 (3)
N1—Fe1—N11	89.86 (4)	Fe1—N1—C1	167.09 (10)
N1^i^—Fe1—N11	90.14 (4)		

Symmetry code: (i) 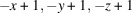 .

**Table 3:** Hydrogen-bond geometry (Å, °) for **1** [Chem scheme1]

*D*—H⋯*A*	*D*—H	H⋯*A*	*D*⋯*A*	*D*—H⋯*A*
C11—H11⋯N1	0.95	2.63	3.190 (3)	118
C15—H15⋯N2	0.95	2.58	3.113 (3)	115
C21—H21⋯N2	0.95	2.54	3.108 (3)	118
C24—H24⋯N22^i^	0.95	2.67	3.514 (4)	148
C25—H25⋯N1	0.95	2.61	3.181 (3)	119
C31—H31⋯N1	0.95	2.67	3.214 (3)	117
C35—H35⋯N2	0.95	2.53	3.091 (3)	118
C35—H35⋯N12^ii^	0.95	2.67	3.538 (4)	151
C41—H41⋯N22^iii^	0.95	2.61	3.487 (3)	154
C44—H44⋯S1^iv^	0.95	2.82	3.498 (3)	129
C45—H45⋯N2	0.95	2.55	3.123 (3)	119

Symmetry codes: (i) 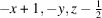 ; (ii) 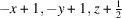 ; (iii) 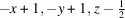 ; (iv) 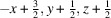 .

**Table 4:** Hydrogen-bond geometry (Å, °) for **2** [Chem scheme1]

*D*—H⋯*A*	*D*—H	H⋯*A*	*D*⋯*A*	*D*—H⋯*A*
O1—H1*A*⋯N21	0.89 (2)	1.88 (2)	2.7615 (14)	175 (2)
O1—H1*B*⋯S1^ii^	0.81 (2)	2.62 (2)	3.3184 (9)	145.7 (18)
C11—H11⋯N1	0.95	2.54	3.1243 (16)	120
C11—H11⋯S1^iii^	0.95	3.03	3.6833 (12)	128
C14—H14⋯S1^iv^	0.95	2.98	3.7688 (13)	141
C15—H15⋯N1^i^	0.95	2.67	3.1894 (16)	115
C21—H21⋯S1	0.95	2.92	3.8513 (13)	165
C24—H24⋯N22^ii^	0.95	2.67	3.3082 (17)	125
C25—H25⋯S1^ii^	0.95	3.01	3.8056 (13)	142

Symmetry codes: (i) 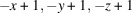 ; (ii) 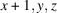 ; (iii) 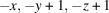 ; (iv) 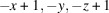 .

**Table 5:** Experimental details

	**1**	**2**
Crystal data		
Chemical formula	[Fe(NCS)_2_(C_6_H_4_N_2_)_4_]	[Fe(NCS)_2_(C_6_H_4_N_2_)_2_(H_2_O)_2_]·2C_6_H_4_N_2_
*M*_r_	588.46	624.49
Crystal system, space group	Orthorhombic, *P* *n* *a*2_1_	Triclinic, *P* 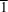
Temperature (K)	100	100
*a*, *b*, *c* (Å)	20.3549 (2), 10.2084 (1), 13.0310 (1)	8.1065 (1), 8.2880 (1), 11.4347 (2)
α, β, γ (°)	90, 90, 90	84.765 (1), 77.787 (1), 70.826 (1)
*V* (Å^3^)	2707.72 (4)	709.02 (2)
*Z*	4	1
Radiation type	Cu *K*α	Cu *K*α
μ (mm^−1^)	6.21	6.01
Crystal size (mm)	0.10 × 0.08 × 0.06	0.11 × 0.10 × 0.08

Data collection		
Diffractometer	XtaLAB Synergy, Dualflex, HyPix	XtaLAB Synergy, Dualflex, HyPix
Absorption correction	Multi-scan (*CrysAlis PRO*; Rigaku OD, 2023 ▸)	Multi-scan (*CrysAlis PRO*; Rigaku OD, 2023 ▸)
*T*_min_, *T*_max_	0.745, 1.000	0.727, 1.000
No. of measured, independent and observed [*I* > 2σ(*I*)] reflections	26794, 5727, 5676	29397, 2999, 2999
*R*_int_	0.019	0.022
(sin θ/λ)_max_ (Å^−1^)	0.639	0.639

Refinement		
*R*[*F* ^2^ > 2σ(*F* ^2^)], *wR*(*F* ^2^), *S*	0.027, 0.074, 1.06	0.022, 0.060, 1.15
No. of reflections	5727	2999
No. of parameters	352	196
No. of restraints	1	0
H-atom treatment	H-atom parameters constrained	H atoms treated by a mixture of independent and constrained refinement
Δρ_max_, Δρ_min_ (e Å^−3^)	0.24, −0.29	0.29, −0.25
Absolute structure	Classical Flack method preferred over Parsons because s.u. lower	–
Absolute structure parameter	−0.001 (3)	–

Computer programs: *CrysAlis PRO* (Rigaku OD, 2023 ▸), *SHELXT2014/5* (Sheldrick, 2015*b* ▸), *SHELXL2016/6* (Sheldrick, 2015*a* ▸), *DIAMOND* (Brandenburg & Putz, 1999 ▸) and *publCIF* (Westrip, 2010 ▸).

## supplementary crystallographic information

### Tetrakis(pyridine-3-carbonitrile)dithiocyanatoiron(II) (1) Crystal data

**Table:**

[Fe(NCS)_2_(C_6_H_4_N_2_)_4_]	*D*_x_ = 1.444 Mg m^−^^3^
*M**_r_* = 588.46	Cu *K*α radiation, λ = 1.54178 Å
Orthorhombic, *P**n**a*2_1_	Cell parameters from 22008 reflections
*a* = 20.3549 (2) Å	θ = 4.3–79.7°
*b* = 10.2084 (1) Å	µ = 6.21 mm^−^^1^
*c* = 13.0310 (1) Å	*T* = 100 K
*V* = 2707.72 (4) Å^3^	Block, yellow
*Z* = 4	0.10 × 0.08 × 0.06 mm
*F*(000) = 1200	

### Tetrakis(pyridine-3-carbonitrile)dithiocyanatoiron(II) (1) Data collection

**Table:**

XtaLAB Synergy, Dualflex, HyPix diffractometer	5727 independent reflections
Radiation source: micro-focus sealed X-ray tube, PhotonJet (Cu) X-ray Source	5676 reflections with *I* > 2σ(*I*)
Mirror monochromator	*R*_int_ = 0.019
Detector resolution: 10.0000 pixels mm^-1^	θ_max_ = 80.1°, θ_min_ = 4.3°
ω scans	*h* = −25→24
Absorption correction: multi-scan (CrysalisPro; Rigaku OD, 2023)	*k* = −13→13
*T*_min_ = 0.745, *T*_max_ = 1.000	*l* = −16→15
26794 measured reflections	

### Tetrakis(pyridine-3-carbonitrile)dithiocyanatoiron(II) (1) Refinement

**Table:**

Refinement on *F*^2^	Hydrogen site location: inferred from neighbouring sites
Least-squares matrix: full	H-atom parameters constrained
*R*[*F*^2^ > 2σ(*F*^2^)] = 0.027	*w* = 1/[σ^2^(*F*_o_^2^) + (0.0521*P*)^2^ + 0.7383*P*] where *P* = (*F*_o_^2^ + 2*F*_c_^2^)/3
*wR*(*F*^2^) = 0.074	(Δ/σ)_max_ = 0.001
*S* = 1.06	Δρ_max_ = 0.24 e Å^−^^3^
5727 reflections	Δρ_min_ = −0.29 e Å^−^^3^
352 parameters	Absolute structure: Classical Flack method preferred over Parsons because s.u. lower
1 restraint	Absolute structure parameter: −0.001 (3)
Primary atom site location: dual	

### Tetrakis(pyridine-3-carbonitrile)dithiocyanatoiron(II) (1) Special details

**Table:**

Geometry. All esds (except the esd in the dihedral angle between two l.s. planes) are estimated using the full covariance matrix. The cell esds are taken into account individually in the estimation of esds in distances, angles and torsion angles; correlations between esds in cell parameters are only used when they are defined by crystal symmetry. An approximate (isotropic) treatment of cell esds is used for estimating esds involving l.s. planes.

### Tetrakis(pyridine-3-carbonitrile)dithiocyanatoiron(II) (1) Fractional atomic coordinates and isotropic or equivalent isotropic displacement parameters (Å^2^)

**Table:**

	*x*	*y*	*z*	*U*_iso_*/*U*_eq_	
Fe1	0.61644 (2)	0.57715 (4)	0.50208 (3)	0.01625 (10)	
N1	0.60947 (11)	0.5803 (2)	0.34404 (18)	0.0220 (5)	
C1	0.60437 (12)	0.5722 (2)	0.2549 (2)	0.0182 (5)	
S1	0.59757 (3)	0.56051 (6)	0.13118 (5)	0.02232 (13)	
N2	0.62205 (11)	0.5735 (2)	0.66062 (18)	0.0207 (5)	
C2	0.61060 (12)	0.5872 (2)	0.7474 (2)	0.0184 (5)	
S2	0.59268 (4)	0.60626 (6)	0.86779 (5)	0.02877 (15)	
N11	0.51976 (9)	0.68579 (19)	0.51593 (16)	0.0186 (4)	
C11	0.47464 (12)	0.6794 (2)	0.44172 (17)	0.0193 (4)	
H11	0.485160	0.634024	0.380244	0.023*	
C12	0.41249 (12)	0.7369 (2)	0.45077 (19)	0.0203 (5)	
C13	0.39634 (12)	0.8027 (2)	0.5405 (2)	0.0218 (5)	
H13	0.354112	0.840674	0.549291	0.026*	
C14	0.44349 (12)	0.8114 (2)	0.61682 (19)	0.0227 (5)	
H14	0.434368	0.856933	0.678783	0.027*	
C15	0.50423 (12)	0.7527 (2)	0.60161 (18)	0.0214 (5)	
H15	0.536372	0.760156	0.654147	0.026*	
C16	0.36587 (13)	0.7271 (2)	0.3681 (2)	0.0233 (5)	
N12	0.32775 (12)	0.7231 (2)	0.30298 (19)	0.0312 (5)	
N21	0.57491 (9)	0.37054 (19)	0.50576 (17)	0.0190 (4)	
C21	0.54990 (11)	0.3198 (2)	0.59197 (19)	0.0198 (4)	
H21	0.547628	0.373602	0.651370	0.024*	
C22	0.52700 (11)	0.1912 (2)	0.59825 (19)	0.0210 (5)	
C23	0.53071 (12)	0.1103 (2)	0.5122 (2)	0.0244 (5)	
H23	0.516095	0.021992	0.514719	0.029*	
C24	0.55633 (13)	0.1629 (2)	0.4232 (2)	0.0258 (5)	
H24	0.559409	0.111195	0.362787	0.031*	
C25	0.57755 (13)	0.2922 (2)	0.4227 (2)	0.0231 (5)	
H25	0.594798	0.327145	0.360763	0.028*	
C26	0.49712 (13)	0.1461 (3)	0.6918 (2)	0.0250 (5)	
N22	0.47136 (13)	0.1104 (2)	0.7652 (2)	0.0325 (5)	
N31	0.71545 (9)	0.4783 (2)	0.49342 (16)	0.0208 (4)	
C31	0.75413 (12)	0.4869 (2)	0.4104 (2)	0.0219 (5)	
H31	0.738616	0.533481	0.352142	0.026*	
C32	0.81617 (13)	0.4300 (2)	0.4066 (2)	0.0237 (5)	
C33	0.84021 (12)	0.3634 (3)	0.4921 (2)	0.0275 (5)	
H33	0.882745	0.325046	0.491573	0.033*	
C34	0.80029 (14)	0.3547 (3)	0.5778 (2)	0.0293 (5)	
H34	0.815036	0.310288	0.637584	0.035*	
C35	0.73825 (13)	0.4119 (2)	0.5752 (2)	0.0248 (5)	
H35	0.710769	0.403611	0.633745	0.030*	
C36	0.85443 (14)	0.4395 (3)	0.3136 (2)	0.0285 (6)	
N32	0.88400 (12)	0.4469 (3)	0.2389 (2)	0.0368 (6)	
N41	0.67037 (9)	0.76769 (19)	0.49903 (17)	0.0190 (4)	
C41	0.66633 (11)	0.8538 (2)	0.42188 (19)	0.0212 (4)	
H41	0.635285	0.838771	0.368733	0.025*	
C42	0.70611 (12)	0.9649 (3)	0.4167 (2)	0.0220 (5)	
C43	0.75141 (12)	0.9892 (3)	0.4943 (2)	0.0263 (5)	
H43	0.779162	1.064013	0.492090	0.032*	
C44	0.75473 (13)	0.9008 (3)	0.5748 (2)	0.0273 (5)	
H44	0.784604	0.914585	0.629764	0.033*	
C45	0.71398 (12)	0.7922 (3)	0.5742 (2)	0.0229 (5)	
H45	0.716965	0.731957	0.629611	0.027*	
C46	0.70086 (13)	1.0523 (3)	0.3298 (2)	0.0260 (5)	
N42	0.69645 (12)	1.1210 (3)	0.2610 (2)	0.0350 (6)	

### Tetrakis(pyridine-3-carbonitrile)dithiocyanatoiron(II) (1) Atomic displacement parameters (Å^2^)

**Table:**

	*U*^11^	*U*^22^	*U*^33^	*U*^12^	*U*^13^	*U*^23^
Fe1	0.01820 (17)	0.01806 (17)	0.01250 (17)	−0.00105 (13)	0.00061 (15)	0.00084 (13)
N1	0.0273 (11)	0.0241 (11)	0.0147 (12)	0.0001 (8)	−0.0005 (8)	0.0004 (7)
C1	0.0154 (10)	0.0165 (11)	0.0225 (15)	0.0006 (8)	0.0018 (9)	0.0016 (9)
S1	0.0246 (3)	0.0278 (3)	0.0145 (3)	0.0040 (2)	−0.0001 (2)	−0.0018 (2)
N2	0.0233 (10)	0.0220 (11)	0.0167 (11)	−0.0001 (8)	−0.0013 (7)	0.0010 (7)
C2	0.0208 (11)	0.0159 (11)	0.0185 (14)	−0.0020 (8)	−0.0029 (9)	0.0024 (9)
S2	0.0467 (4)	0.0235 (3)	0.0162 (3)	−0.0019 (3)	0.0055 (3)	−0.0007 (3)
N11	0.0195 (8)	0.0169 (9)	0.0196 (10)	−0.0021 (7)	0.0008 (7)	0.0002 (7)
C11	0.0222 (11)	0.0183 (10)	0.0175 (11)	−0.0019 (8)	0.0008 (8)	0.0011 (8)
C12	0.0210 (11)	0.0187 (11)	0.0210 (12)	−0.0023 (8)	−0.0002 (9)	0.0008 (9)
C13	0.0233 (11)	0.0177 (11)	0.0243 (12)	−0.0001 (9)	0.0002 (9)	0.0006 (9)
C14	0.0286 (12)	0.0189 (11)	0.0206 (12)	0.0012 (9)	−0.0004 (9)	−0.0040 (9)
C15	0.0251 (11)	0.0193 (10)	0.0199 (11)	−0.0022 (9)	−0.0021 (9)	−0.0013 (9)
C16	0.0260 (12)	0.0195 (11)	0.0245 (12)	0.0018 (9)	−0.0002 (10)	0.0001 (10)
N12	0.0324 (12)	0.0298 (11)	0.0314 (13)	0.0020 (10)	−0.0101 (10)	−0.0030 (9)
N21	0.0187 (8)	0.0200 (9)	0.0182 (9)	−0.0009 (7)	0.0006 (7)	0.0000 (8)
C21	0.0197 (10)	0.0197 (11)	0.0201 (11)	−0.0005 (8)	0.0015 (8)	−0.0020 (9)
C22	0.0197 (10)	0.0203 (11)	0.0230 (12)	0.0006 (8)	0.0039 (9)	0.0018 (9)
C23	0.0264 (11)	0.0193 (11)	0.0276 (13)	−0.0010 (9)	0.0038 (10)	−0.0011 (10)
C24	0.0327 (13)	0.0204 (12)	0.0244 (12)	−0.0013 (10)	0.0036 (10)	−0.0037 (10)
C25	0.0284 (12)	0.0219 (12)	0.0191 (11)	0.0001 (9)	0.0037 (9)	0.0001 (9)
C26	0.0284 (12)	0.0185 (11)	0.0280 (13)	−0.0004 (9)	0.0060 (10)	−0.0019 (10)
N22	0.0417 (14)	0.0225 (11)	0.0333 (13)	−0.0015 (9)	0.0141 (10)	0.0011 (9)
N31	0.0208 (9)	0.0207 (9)	0.0210 (10)	0.0006 (7)	0.0005 (8)	0.0005 (8)
C31	0.0219 (11)	0.0206 (11)	0.0232 (11)	−0.0020 (9)	0.0021 (9)	−0.0010 (9)
C32	0.0223 (12)	0.0216 (12)	0.0274 (14)	−0.0017 (9)	0.0038 (10)	−0.0050 (9)
C33	0.0246 (11)	0.0255 (11)	0.0324 (14)	0.0068 (10)	−0.0025 (10)	−0.0063 (10)
C34	0.0328 (13)	0.0287 (13)	0.0263 (13)	0.0102 (11)	−0.0037 (10)	−0.0002 (11)
C35	0.0278 (12)	0.0248 (11)	0.0219 (12)	0.0048 (10)	0.0026 (10)	−0.0001 (10)
C36	0.0234 (13)	0.0257 (12)	0.0363 (15)	0.0005 (9)	0.0058 (11)	−0.0049 (11)
N32	0.0333 (13)	0.0315 (12)	0.0456 (16)	0.0010 (9)	0.0146 (11)	−0.0031 (11)
N41	0.0182 (8)	0.0193 (9)	0.0196 (8)	−0.0012 (7)	−0.0011 (8)	0.0018 (8)
C41	0.0195 (10)	0.0224 (11)	0.0217 (11)	0.0011 (9)	−0.0003 (9)	0.0011 (10)
C42	0.0212 (10)	0.0228 (11)	0.0221 (12)	0.0003 (9)	0.0037 (9)	0.0042 (10)
C43	0.0231 (11)	0.0271 (12)	0.0288 (13)	−0.0069 (9)	0.0007 (10)	0.0023 (10)
C44	0.0240 (12)	0.0329 (13)	0.0250 (13)	−0.0080 (10)	−0.0047 (10)	0.0025 (11)
C45	0.0230 (11)	0.0242 (11)	0.0215 (11)	−0.0018 (9)	−0.0030 (9)	0.0028 (10)
C46	0.0241 (12)	0.0241 (12)	0.0297 (14)	−0.0011 (9)	0.0041 (10)	0.0039 (11)
N42	0.0339 (12)	0.0356 (13)	0.0356 (14)	−0.0006 (10)	0.0048 (10)	0.0130 (11)

### Tetrakis(pyridine-3-carbonitrile)dithiocyanatoiron(II) (1) Geometric parameters (Å, º)

**Table:**

Fe1—N1	2.065 (2)	C23—C24	1.380 (4)
Fe1—N2	2.069 (2)	C24—H24	0.9500
Fe1—N11	2.2660 (19)	C24—C25	1.389 (4)
Fe1—N21	2.273 (2)	C25—H25	0.9500
Fe1—N31	2.257 (2)	C26—N22	1.149 (4)
Fe1—N41	2.2339 (19)	N31—C31	1.341 (3)
N1—C1	1.169 (4)	N31—C35	1.346 (3)
C1—S1	1.622 (3)	C31—H31	0.9500
N2—C2	1.163 (4)	C31—C32	1.391 (4)
C2—S2	1.622 (3)	C32—C33	1.394 (4)
N11—C11	1.335 (3)	C32—C36	1.444 (4)
N11—C15	1.347 (3)	C33—H33	0.9500
C11—H11	0.9500	C33—C34	1.384 (4)
C11—C12	1.399 (3)	C34—H34	0.9500
C12—C13	1.388 (3)	C34—C35	1.392 (4)
C12—C16	1.439 (3)	C35—H35	0.9500
C13—H13	0.9500	C36—N32	1.146 (4)
C13—C14	1.385 (4)	N41—C41	1.338 (3)
C14—H14	0.9500	N41—C45	1.345 (3)
C14—C15	1.388 (3)	C41—H41	0.9500
C15—H15	0.9500	C41—C42	1.395 (4)
C16—N12	1.151 (4)	C42—C43	1.390 (4)
N21—C21	1.338 (3)	C42—C46	1.447 (4)
N21—C25	1.346 (3)	C43—H43	0.9500
C21—H21	0.9500	C43—C44	1.386 (4)
C21—C22	1.396 (3)	C44—H44	0.9500
C22—C23	1.395 (3)	C44—C45	1.385 (4)
C22—C26	1.438 (3)	C45—H45	0.9500
C23—H23	0.9500	C46—N42	1.141 (4)
			
N1—Fe1—N2	179.21 (9)	C24—C23—C22	117.7 (2)
N1—Fe1—N11	90.70 (8)	C24—C23—H23	121.1
N1—Fe1—N21	90.56 (8)	C23—C24—H24	120.3
N1—Fe1—N31	91.05 (8)	C23—C24—C25	119.4 (2)
N1—Fe1—N41	90.15 (9)	C25—C24—H24	120.3
N2—Fe1—N11	88.70 (8)	N21—C25—C24	123.3 (2)
N2—Fe1—N21	89.00 (8)	N21—C25—H25	118.4
N2—Fe1—N31	89.57 (8)	C24—C25—H25	118.4
N2—Fe1—N41	90.37 (8)	N22—C26—C22	177.9 (3)
N11—Fe1—N21	97.44 (7)	C31—N31—Fe1	122.38 (17)
N31—Fe1—N11	176.72 (7)	C31—N31—C35	118.0 (2)
N31—Fe1—N21	85.31 (7)	C35—N31—Fe1	119.59 (17)
N41—Fe1—N11	90.12 (7)	N31—C31—H31	118.8
N41—Fe1—N21	172.40 (7)	N31—C31—C32	122.3 (2)
N41—Fe1—N31	87.11 (7)	C32—C31—H31	118.8
Fe1—N1—C1	175.0 (2)	C31—C32—C33	119.6 (2)
N1—C1—S1	179.7 (3)	C31—C32—C36	119.4 (3)
Fe1—N2—C2	163.2 (2)	C33—C32—C36	121.0 (2)
N2—C2—S2	178.6 (2)	C32—C33—H33	121.0
C11—N11—Fe1	121.06 (16)	C34—C33—C32	118.0 (2)
C11—N11—C15	117.6 (2)	C34—C33—H33	121.0
C15—N11—Fe1	121.22 (16)	C33—C34—H34	120.5
N11—C11—H11	118.6	C33—C34—C35	119.1 (3)
N11—C11—C12	122.7 (2)	C35—C34—H34	120.5
C12—C11—H11	118.6	N31—C35—C34	122.9 (3)
C11—C12—C16	120.3 (2)	N31—C35—H35	118.5
C13—C12—C11	119.2 (2)	C34—C35—H35	118.5
C13—C12—C16	120.5 (2)	N32—C36—C32	179.0 (3)
C12—C13—H13	120.9	C41—N41—Fe1	123.72 (16)
C14—C13—C12	118.2 (2)	C41—N41—C45	117.8 (2)
C14—C13—H13	120.9	C45—N41—Fe1	118.22 (16)
C13—C14—H14	120.4	N41—C41—H41	118.8
C13—C14—C15	119.1 (2)	N41—C41—C42	122.3 (2)
C15—C14—H14	120.4	C42—C41—H41	118.8
N11—C15—C14	123.1 (2)	C41—C42—C46	119.7 (2)
N11—C15—H15	118.4	C43—C42—C41	119.7 (2)
C14—C15—H15	118.4	C43—C42—C46	120.5 (2)
N12—C16—C12	177.8 (3)	C42—C43—H43	121.1
C21—N21—Fe1	121.22 (16)	C44—C43—C42	117.8 (2)
C21—N21—C25	117.4 (2)	C44—C43—H43	121.1
C25—N21—Fe1	121.28 (17)	C43—C44—H44	120.4
N21—C21—H21	118.6	C45—C44—C43	119.2 (2)
N21—C21—C22	122.7 (2)	C45—C44—H44	120.4
C22—C21—H21	118.6	N41—C45—C44	123.2 (2)
C21—C22—C26	119.5 (2)	N41—C45—H45	118.4
C23—C22—C21	119.5 (2)	C44—C45—H45	118.4
C23—C22—C26	121.0 (2)	N42—C46—C42	179.7 (3)
C22—C23—H23	121.1		

### Tetrakis(pyridine-3-carbonitrile)dithiocyanatoiron(II) (1) Hydrogen-bond geometry (Å, º)

**Table:**

*D*—H···*A*	*D*—H	H···*A*	*D*···*A*	*D*—H···*A*
C11—H11···N1	0.95	2.63	3.190 (3)	118
C15—H15···N2	0.95	2.58	3.113 (3)	115
C21—H21···N2	0.95	2.54	3.108 (3)	118
C24—H24···N22^i^	0.95	2.67	3.514 (4)	148
C25—H25···N1	0.95	2.61	3.181 (3)	119
C31—H31···N1	0.95	2.67	3.214 (3)	117
C35—H35···N2	0.95	2.53	3.091 (3)	118
C35—H35···N12^ii^	0.95	2.67	3.538 (4)	151
C41—H41···N22^iii^	0.95	2.61	3.487 (3)	154
C44—H44···S1^iv^	0.95	2.82	3.498 (3)	129
C45—H45···N2	0.95	2.55	3.123 (3)	119

Symmetry codes: (i) −*x*+1, −*y*, *z*−1/2; (ii) −*x*+1, −*y*+1, *z*+1/2; (iii) −*x*+1, −*y*+1, *z*−1/2; (iv) −*x*+3/2, *y*+1/2, *z*+1/2.

### Diaquabis(pyridine-3-carbonitrile)dithiocyanatoiron(II) pyridine-3-carbonitrile monosolvate (2) Crystal data

**Table:**

[Fe(NCS)_2_(C_6_H_4_N_2_)_2_(H_2_O)_2_]·2C_6_H_4_N_2_	*Z* = 1
*M**_r_* = 624.49	*F*(000) = 320
Triclinic, *P*1	*D*_x_ = 1.463 Mg m^−^^3^
*a* = 8.1065 (1) Å	Cu *K*α radiation, λ = 1.54184 Å
*b* = 8.2880 (1) Å	Cell parameters from 25131 reflections
*c* = 11.4347 (2) Å	θ = 4.0–79.7°
α = 84.765 (1)°	µ = 6.01 mm^−^^1^
β = 77.787 (1)°	*T* = 100 K
γ = 70.826 (1)°	Block, yellow
*V* = 709.02 (2) Å^3^	0.11 × 0.10 × 0.08 mm

### Diaquabis(pyridine-3-carbonitrile)dithiocyanatoiron(II) pyridine-3-carbonitrile monosolvate (2) Data collection

**Table:**

XtaLAB Synergy, Dualflex, HyPix diffractometer	2999 independent reflections
Radiation source: micro-focus sealed X-ray tube, PhotonJet (Cu) X-ray Source	2999 reflections with *I* > 2σ(*I*)
Mirror monochromator	*R*_int_ = 0.022
Detector resolution: 10.0000 pixels mm^-1^	θ_max_ = 80.4°, θ_min_ = 4.0°
ω scans	*h* = −10→10
Absorption correction: multi-scan (CrysalisPro; Rigaku OD, 2023)	*k* = −10→10
*T*_min_ = 0.727, *T*_max_ = 1.000	*l* = −11→14
29397 measured reflections	

### Diaquabis(pyridine-3-carbonitrile)dithiocyanatoiron(II) pyridine-3-carbonitrile monosolvate (2) Refinement

**Table:**

Refinement on *F*^2^	Hydrogen site location: mixed
Least-squares matrix: full	H atoms treated by a mixture of independent and constrained refinement
*R*[*F*^2^ > 2σ(*F*^2^)] = 0.022	*w* = 1/[σ^2^(*F*_o_^2^) + (0.0302*P*)^2^ + 0.2457*P*] where *P* = (*F*_o_^2^ + 2*F*_c_^2^)/3
*wR*(*F*^2^) = 0.060	(Δ/σ)_max_ = 0.001
*S* = 1.15	Δρ_max_ = 0.29 e Å^−^^3^
2999 reflections	Δρ_min_ = −0.24 e Å^−^^3^
196 parameters	Extinction correction: SHELXL-2016/6 (Sheldrick 2016), Fc^*^=kFc[1+0.001xFc^2^λ^3^/sin(2θ)]^-1/4^
0 restraints	Extinction coefficient: 0.0036 (4)
Primary atom site location: dual	

### Diaquabis(pyridine-3-carbonitrile)dithiocyanatoiron(II) pyridine-3-carbonitrile monosolvate (2) Special details

**Table:**

Geometry. All esds (except the esd in the dihedral angle between two l.s. planes) are estimated using the full covariance matrix. The cell esds are taken into account individually in the estimation of esds in distances, angles and torsion angles; correlations between esds in cell parameters are only used when they are defined by crystal symmetry. An approximate (isotropic) treatment of cell esds is used for estimating esds involving l.s. planes.

### Diaquabis(pyridine-3-carbonitrile)dithiocyanatoiron(II) pyridine-3-carbonitrile monosolvate (2) Fractional atomic coordinates and isotropic or equivalent isotropic displacement parameters (Å^2^)

**Table:**

	*x*	*y*	*z*	*U*_iso_*/*U*_eq_	
Fe1	0.500000	0.500000	0.500000	0.01216 (8)	
N1	0.23749 (14)	0.50517 (14)	0.58058 (10)	0.0175 (2)	
C1	0.10930 (16)	0.48617 (15)	0.64015 (11)	0.0145 (2)	
S1	−0.06651 (4)	0.45890 (4)	0.72985 (3)	0.01772 (9)	
O1	0.58744 (12)	0.38660 (11)	0.65983 (8)	0.01684 (18)	
N11	0.55002 (13)	0.24567 (12)	0.42347 (9)	0.0137 (2)	
C11	0.41230 (15)	0.20838 (15)	0.39913 (10)	0.0144 (2)	
H11	0.295430	0.284514	0.423936	0.017*	
C12	0.43436 (16)	0.06162 (15)	0.33861 (11)	0.0151 (2)	
C13	0.60473 (17)	−0.05288 (15)	0.30294 (11)	0.0179 (2)	
H13	0.622799	−0.154127	0.262362	0.022*	
C14	0.74656 (16)	−0.01402 (16)	0.32865 (12)	0.0183 (2)	
H14	0.864668	−0.088837	0.306060	0.022*	
C15	0.71430 (16)	0.13553 (15)	0.38781 (11)	0.0163 (2)	
H15	0.812998	0.161414	0.403931	0.020*	
C16	0.27896 (17)	0.03358 (16)	0.31361 (12)	0.0195 (3)	
N12	0.15470 (16)	0.01357 (16)	0.29369 (12)	0.0292 (3)	
N21	0.40361 (14)	0.59653 (13)	0.85071 (9)	0.0177 (2)	
C21	0.22852 (17)	0.62145 (16)	0.88138 (11)	0.0176 (2)	
H21	0.176535	0.564393	0.838477	0.021*	
C22	0.11976 (16)	0.72784 (15)	0.97378 (11)	0.0160 (2)	
C23	0.19423 (17)	0.81232 (16)	1.03776 (11)	0.0177 (2)	
H23	0.122870	0.885180	1.101462	0.021*	
C24	0.37532 (17)	0.78663 (16)	1.00549 (12)	0.0190 (3)	
H24	0.430907	0.842236	1.046636	0.023*	
C25	0.47433 (16)	0.67873 (16)	0.91238 (11)	0.0177 (2)	
H25	0.598501	0.661992	0.891042	0.021*	
C26	−0.06797 (17)	0.74885 (16)	1.00254 (11)	0.0191 (3)	
N22	−0.21762 (15)	0.76797 (16)	1.02557 (11)	0.0256 (3)	
H1A	0.531 (3)	0.450 (3)	0.724 (2)	0.045 (6)*	
H1B	0.693 (3)	0.369 (3)	0.6554 (18)	0.042 (6)*	

### Diaquabis(pyridine-3-carbonitrile)dithiocyanatoiron(II) pyridine-3-carbonitrile monosolvate (2) Atomic displacement parameters (Å^2^)

**Table:**

	*U*^11^	*U*^22^	*U*^33^	*U*^12^	*U*^13^	*U*^23^
Fe1	0.01011 (13)	0.01477 (14)	0.01245 (14)	−0.00513 (10)	−0.00059 (9)	−0.00395 (9)
N1	0.0134 (5)	0.0216 (5)	0.0188 (5)	−0.0079 (4)	0.0004 (4)	−0.0065 (4)
C1	0.0146 (5)	0.0146 (5)	0.0155 (6)	−0.0034 (4)	−0.0054 (4)	−0.0043 (4)
S1	0.01226 (14)	0.02427 (16)	0.01741 (16)	−0.00805 (11)	−0.00037 (10)	−0.00184 (11)
O1	0.0146 (4)	0.0193 (4)	0.0166 (4)	−0.0046 (3)	−0.0025 (3)	−0.0045 (3)
N11	0.0132 (5)	0.0148 (5)	0.0131 (5)	−0.0048 (4)	−0.0013 (4)	−0.0020 (4)
C11	0.0134 (5)	0.0162 (5)	0.0137 (6)	−0.0048 (4)	−0.0023 (4)	−0.0018 (4)
C12	0.0159 (6)	0.0173 (5)	0.0141 (6)	−0.0077 (5)	−0.0025 (4)	−0.0018 (4)
C13	0.0188 (6)	0.0160 (6)	0.0190 (6)	−0.0064 (5)	−0.0004 (5)	−0.0046 (5)
C14	0.0141 (5)	0.0161 (6)	0.0223 (6)	−0.0029 (4)	0.0000 (5)	−0.0038 (5)
C15	0.0132 (5)	0.0192 (6)	0.0172 (6)	−0.0066 (5)	−0.0019 (4)	−0.0013 (5)
C16	0.0188 (6)	0.0182 (6)	0.0217 (6)	−0.0058 (5)	−0.0019 (5)	−0.0074 (5)
N12	0.0209 (6)	0.0302 (6)	0.0397 (7)	−0.0088 (5)	−0.0059 (5)	−0.0151 (5)
N21	0.0180 (5)	0.0207 (5)	0.0139 (5)	−0.0057 (4)	−0.0024 (4)	−0.0019 (4)
C21	0.0191 (6)	0.0207 (6)	0.0153 (6)	−0.0084 (5)	−0.0041 (5)	−0.0019 (5)
C22	0.0152 (6)	0.0189 (6)	0.0152 (6)	−0.0073 (5)	−0.0034 (4)	0.0005 (4)
C23	0.0181 (6)	0.0193 (6)	0.0162 (6)	−0.0068 (5)	−0.0015 (5)	−0.0040 (5)
C24	0.0183 (6)	0.0225 (6)	0.0194 (6)	−0.0097 (5)	−0.0045 (5)	−0.0029 (5)
C25	0.0147 (5)	0.0213 (6)	0.0172 (6)	−0.0066 (5)	−0.0026 (4)	0.0006 (5)
C26	0.0198 (6)	0.0216 (6)	0.0180 (6)	−0.0084 (5)	−0.0037 (5)	−0.0031 (5)
N22	0.0188 (6)	0.0314 (6)	0.0290 (6)	−0.0103 (5)	−0.0041 (5)	−0.0055 (5)

### Diaquabis(pyridine-3-carbonitrile)dithiocyanatoiron(II) pyridine-3-carbonitrile monosolvate (2) Geometric parameters (Å, º)

**Table:**

Fe1—N1^i^	2.1207 (10)	C13—C14	1.3841 (17)
Fe1—N1	2.1207 (10)	C14—H14	0.9500
Fe1—O1^i^	2.1267 (9)	C14—C15	1.3883 (17)
Fe1—O1	2.1267 (9)	C15—H15	0.9500
Fe1—N11	2.2358 (10)	C16—N12	1.1435 (18)
Fe1—N11^i^	2.2358 (10)	N21—C21	1.3380 (16)
N1—C1	1.1649 (17)	N21—C25	1.3436 (16)
C1—S1	1.6387 (12)	C21—H21	0.9500
O1—H1A	0.89 (2)	C21—C22	1.3916 (18)
O1—H1B	0.81 (2)	C22—C23	1.3956 (17)
N11—C11	1.3390 (15)	C22—C26	1.4419 (17)
N11—C15	1.3463 (15)	C23—H23	0.9500
C11—H11	0.9500	C23—C24	1.3844 (17)
C11—C12	1.3960 (16)	C24—H24	0.9500
C12—C13	1.3949 (17)	C24—C25	1.3850 (18)
C12—C16	1.4421 (17)	C25—H25	0.9500
C13—H13	0.9500	C26—N22	1.1456 (17)
			
N1^i^—Fe1—N1	180.0	C13—C12—C16	121.70 (11)
N1—Fe1—O1	89.42 (4)	C12—C13—H13	121.1
N1^i^—Fe1—O1	90.58 (4)	C14—C13—C12	117.77 (11)
N1—Fe1—O1^i^	90.58 (4)	C14—C13—H13	121.1
N1^i^—Fe1—O1^i^	89.42 (4)	C13—C14—H14	120.4
N1—Fe1—N11	89.86 (4)	C13—C14—C15	119.26 (11)
N1^i^—Fe1—N11	90.14 (4)	C15—C14—H14	120.4
N1^i^—Fe1—N11^i^	89.86 (4)	N11—C15—C14	123.23 (11)
N1—Fe1—N11^i^	90.14 (4)	N11—C15—H15	118.4
O1^i^—Fe1—O1	180.0	C14—C15—H15	118.4
O1^i^—Fe1—N11	87.62 (3)	N12—C16—C12	179.12 (14)
O1^i^—Fe1—N11^i^	92.38 (3)	C21—N21—C25	117.81 (11)
O1—Fe1—N11	92.38 (3)	N21—C21—H21	118.8
O1—Fe1—N11^i^	87.62 (3)	N21—C21—C22	122.43 (11)
N11—Fe1—N11^i^	180.0	C22—C21—H21	118.8
Fe1—N1—C1	167.09 (10)	C21—C22—C23	119.45 (11)
N1—C1—S1	177.12 (11)	C21—C22—C26	119.67 (11)
Fe1—O1—H1A	113.2 (14)	C23—C22—C26	120.87 (11)
Fe1—O1—H1B	112.3 (14)	C22—C23—H23	121.0
H1A—O1—H1B	106.9 (19)	C24—C23—C22	117.93 (12)
C11—N11—Fe1	118.64 (8)	C24—C23—H23	121.0
C11—N11—C15	117.74 (10)	C23—C24—H24	120.5
C15—N11—Fe1	123.18 (8)	C23—C24—C25	119.07 (11)
N11—C11—H11	118.8	C25—C24—H24	120.5
N11—C11—C12	122.34 (11)	N21—C25—C24	123.30 (11)
C12—C11—H11	118.8	N21—C25—H25	118.3
C11—C12—C16	118.64 (11)	C24—C25—H25	118.3
C13—C12—C11	119.66 (11)	N22—C26—C22	179.03 (14)
			
Fe1—N11—C11—C12	172.36 (9)	C16—C12—C13—C14	178.74 (12)
Fe1—N11—C15—C14	−172.87 (9)	N21—C21—C22—C23	−0.10 (19)
N11—C11—C12—C13	0.88 (18)	N21—C21—C22—C26	−179.84 (11)
N11—C11—C12—C16	−178.52 (11)	C21—N21—C25—C24	0.17 (18)
C11—N11—C15—C14	−0.63 (18)	C21—C22—C23—C24	0.31 (18)
C11—C12—C13—C14	−0.64 (18)	C22—C23—C24—C25	−0.28 (19)
C12—C13—C14—C15	−0.17 (18)	C23—C24—C25—N21	0.04 (19)
C13—C14—C15—N11	0.84 (19)	C25—N21—C21—C22	−0.14 (18)
C15—N11—C11—C12	−0.24 (17)	C26—C22—C23—C24	−179.96 (12)

Symmetry code: (i) −*x*+1, −*y*+1, −*z*+1.

### Diaquabis(pyridine-3-carbonitrile)dithiocyanatoiron(II) pyridine-3-carbonitrile monosolvate (2) Hydrogen-bond geometry (Å, º)

**Table:**

*D*—H···*A*	*D*—H	H···*A*	*D*···*A*	*D*—H···*A*
O1—H1*A*···N21	0.89 (2)	1.88 (2)	2.7615 (14)	175 (2)
O1—H1*B*···S1^ii^	0.81 (2)	2.62 (2)	3.3184 (9)	145.7 (18)
C11—H11···N1	0.95	2.54	3.1243 (16)	120
C11—H11···S1^iii^	0.95	3.03	3.6833 (12)	128
C14—H14···S1^iv^	0.95	2.98	3.7688 (13)	141
C15—H15···N1^i^	0.95	2.67	3.1894 (16)	115
C21—H21···S1	0.95	2.92	3.8513 (13)	165
C24—H24···N22^ii^	0.95	2.67	3.3082 (17)	125
C25—H25···S1^ii^	0.95	3.01	3.8056 (13)	142

Symmetry codes: (i) −*x*+1, −*y*+1, −*z*+1; (ii) *x*+1, *y*, *z*; (iii) −*x*, −*y*+1, −*z*+1; (iv) −*x*+1, −*y*, −*z*+1.
