# Supplementary figures and images for: Synthesis, crystal structure and properties of tetra­kis­(pyridine-3-carbo­nitrile)­dithio­cyanatoiron(II) and of diaqua­bis­(pyridine-3-carbo­nitrile)­di­thio­cyanatoiron(II) pyridine-3-carbo­nitrile monosolvate
Source: Acta Crystallogr E Crystallogr Commun. 2023 Oct 31;79(Pt 11):1093–9. doi: 10.1107/S205698902300909X (PMC10626964; doi:10.1107/S205698902300909X)

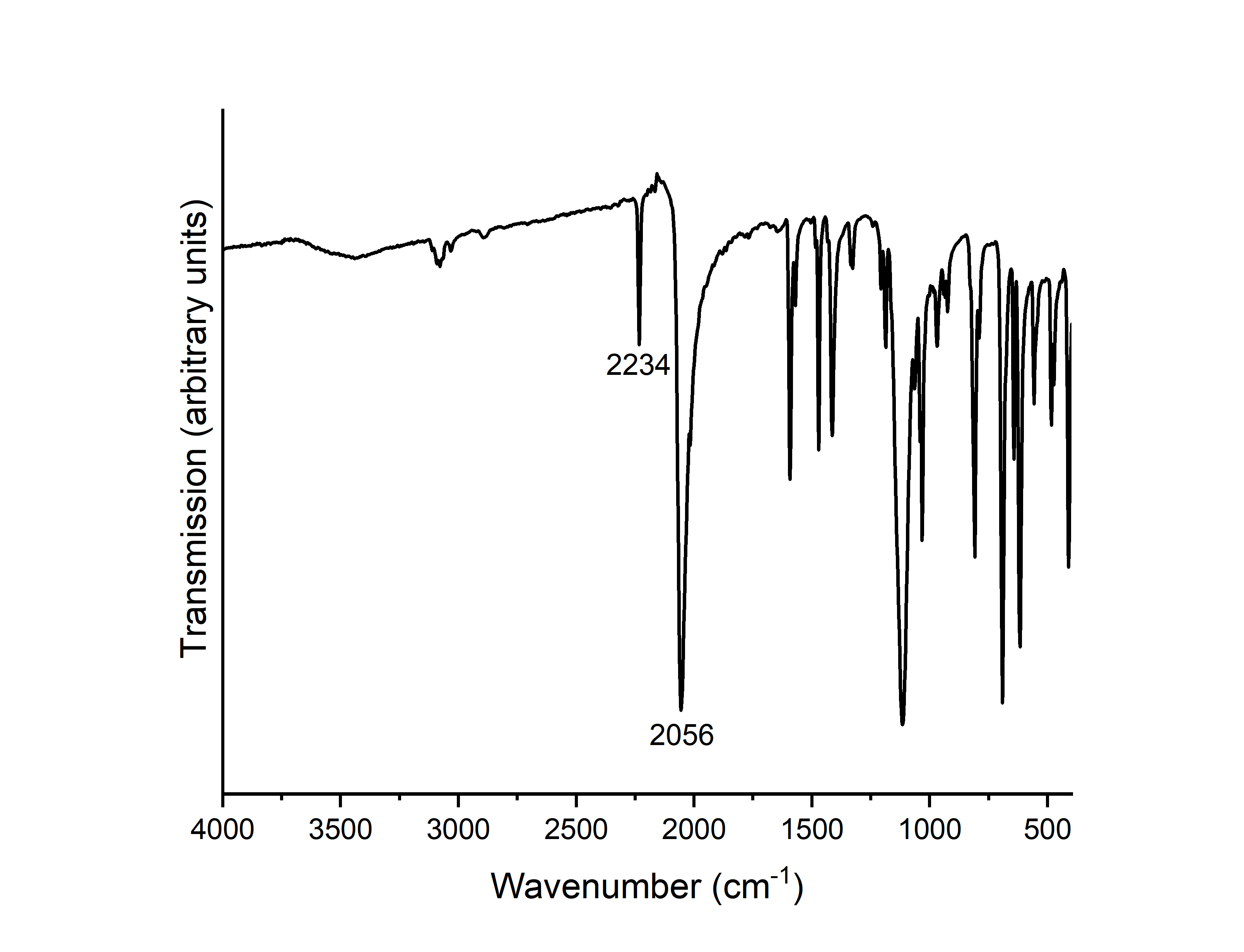

Supplement: Supplementary file 4 [file e-79-01093-sup4.png]

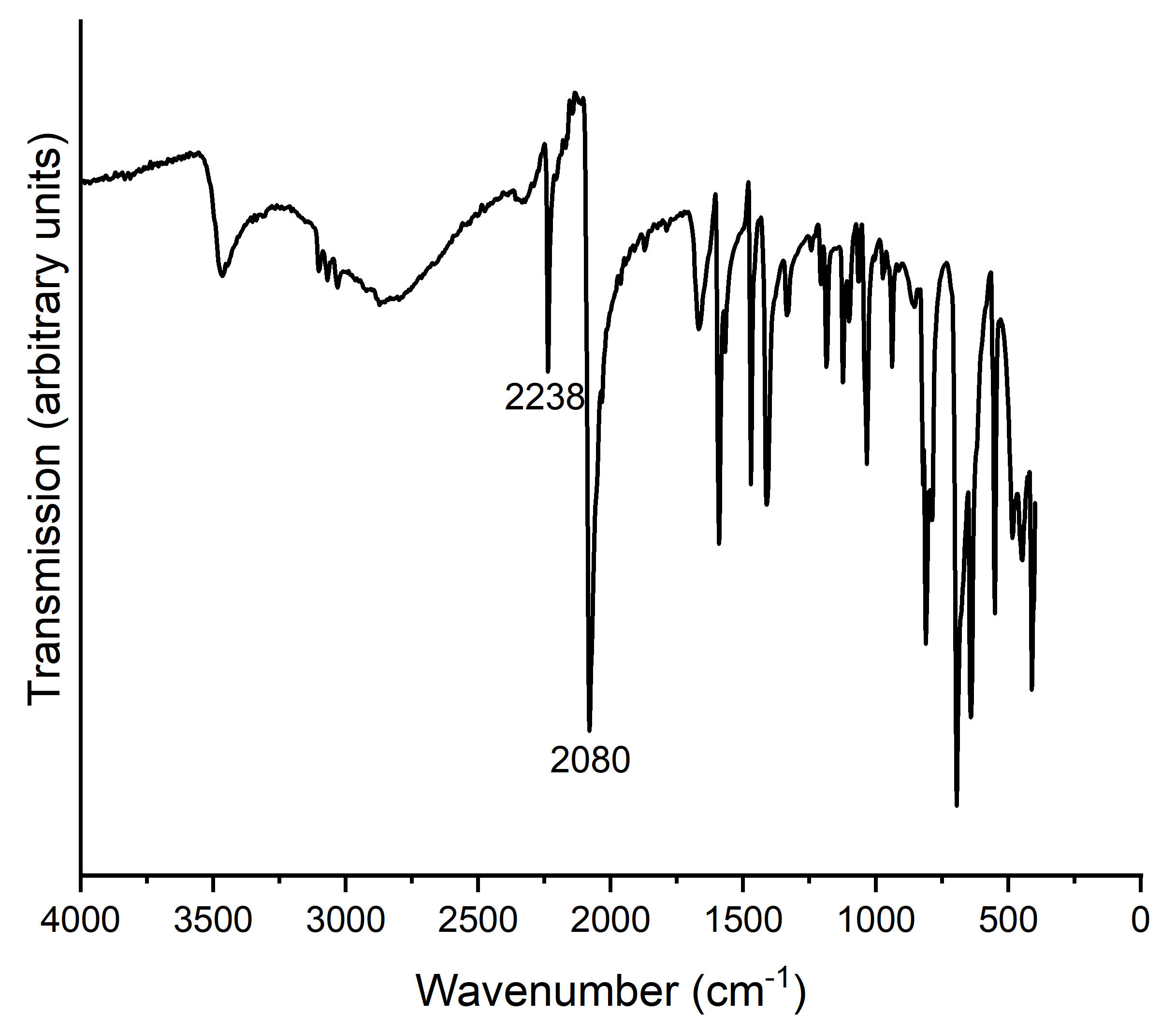

Supplement: Supplementary file 5 [file e-79-01093-sup5.png]

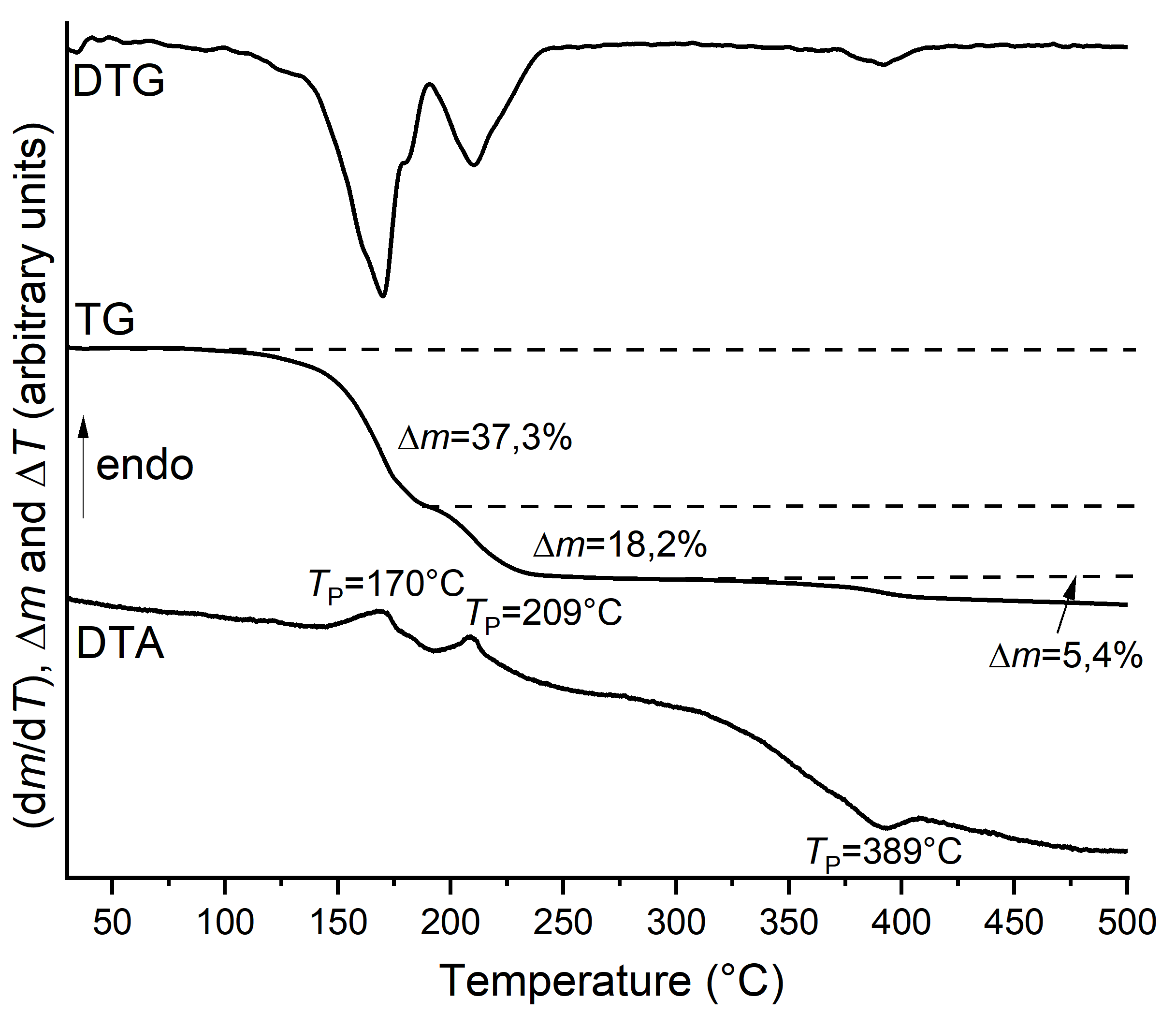

Supplement: Supplementary file 6 [file e-79-01093-sup6.png]

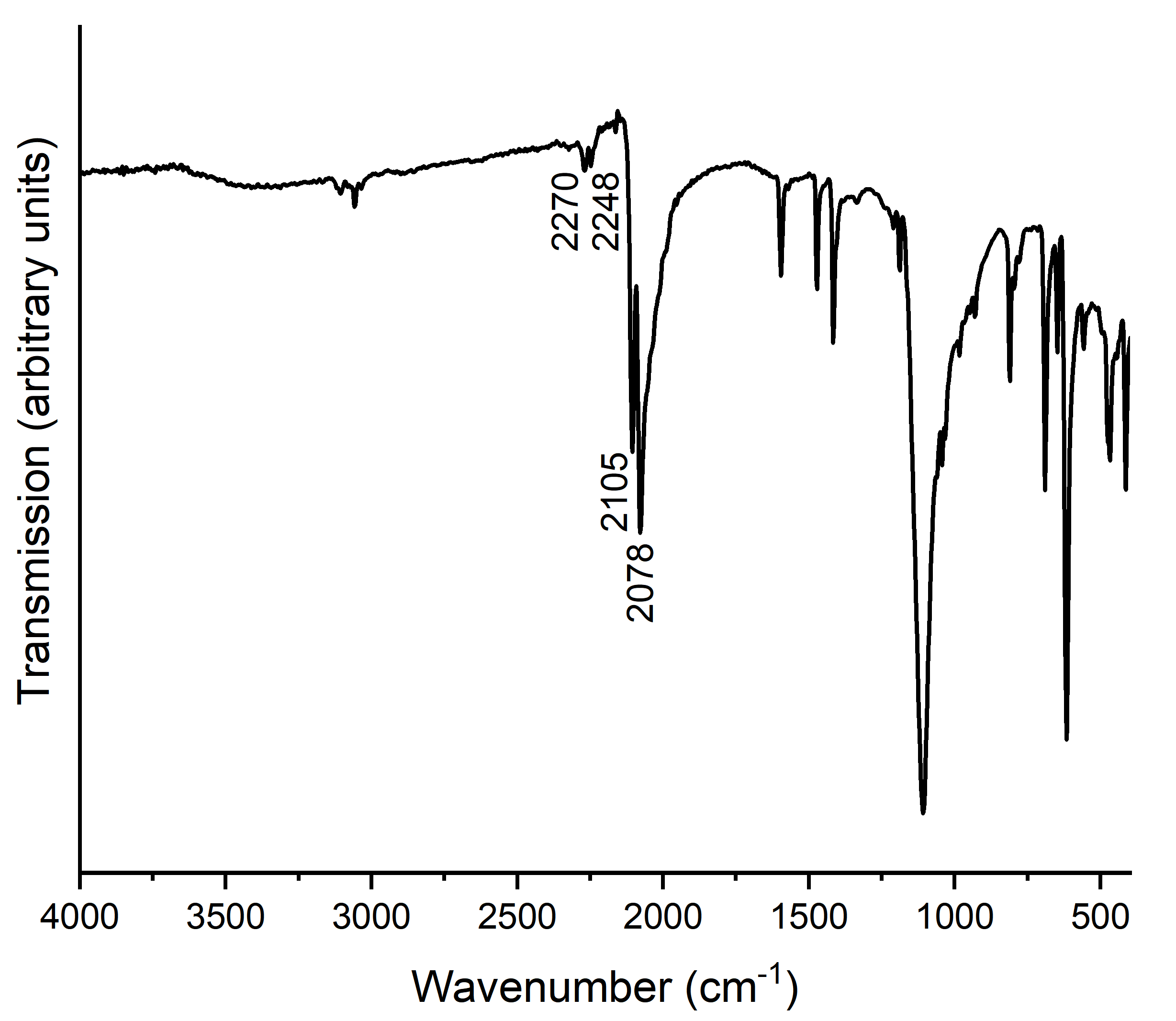

Supplement: Supplementary file 7 [file e-79-01093-sup7.png]

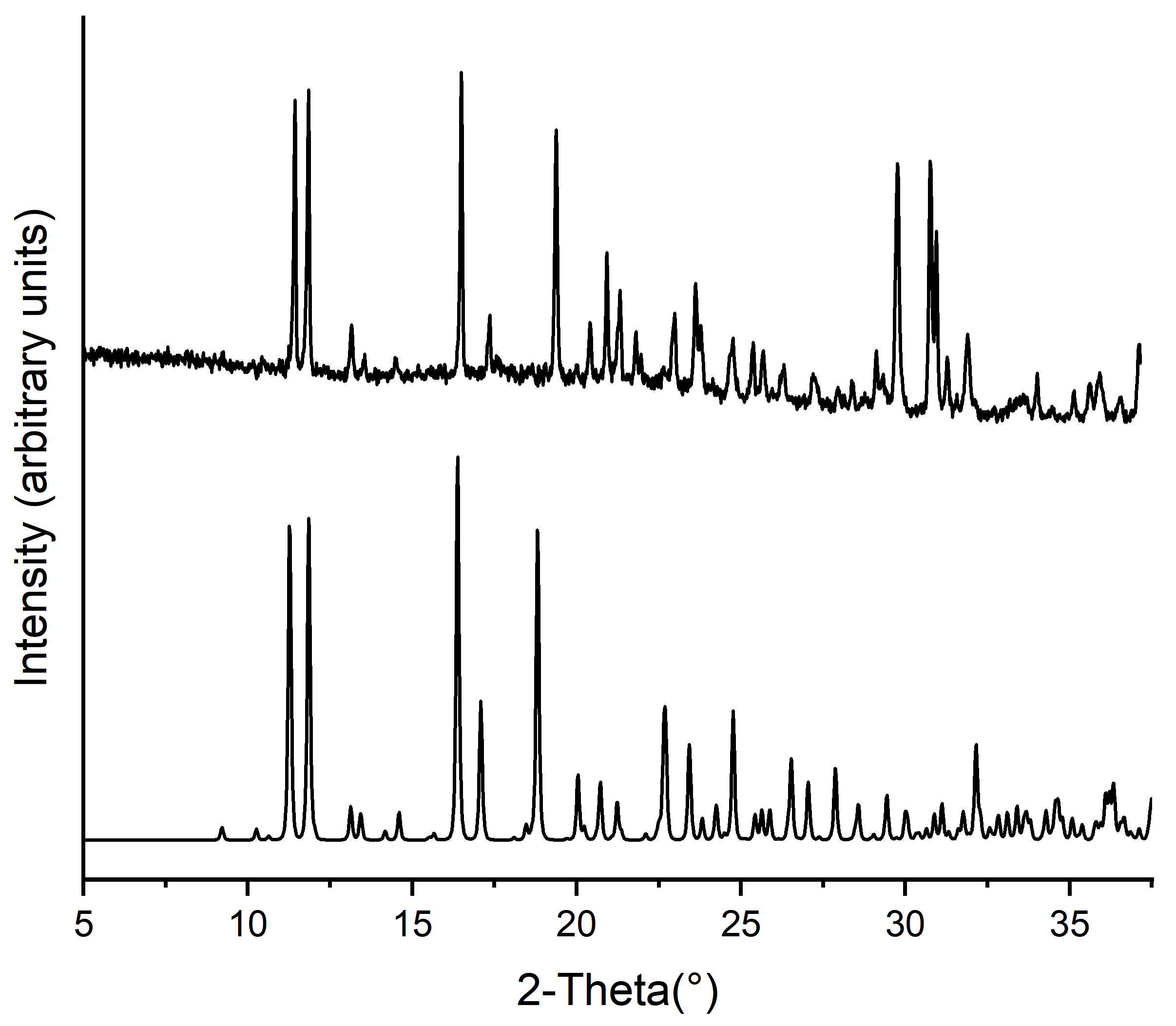

Supplement: Supplementary file 8 [file e-79-01093-sup8.png]

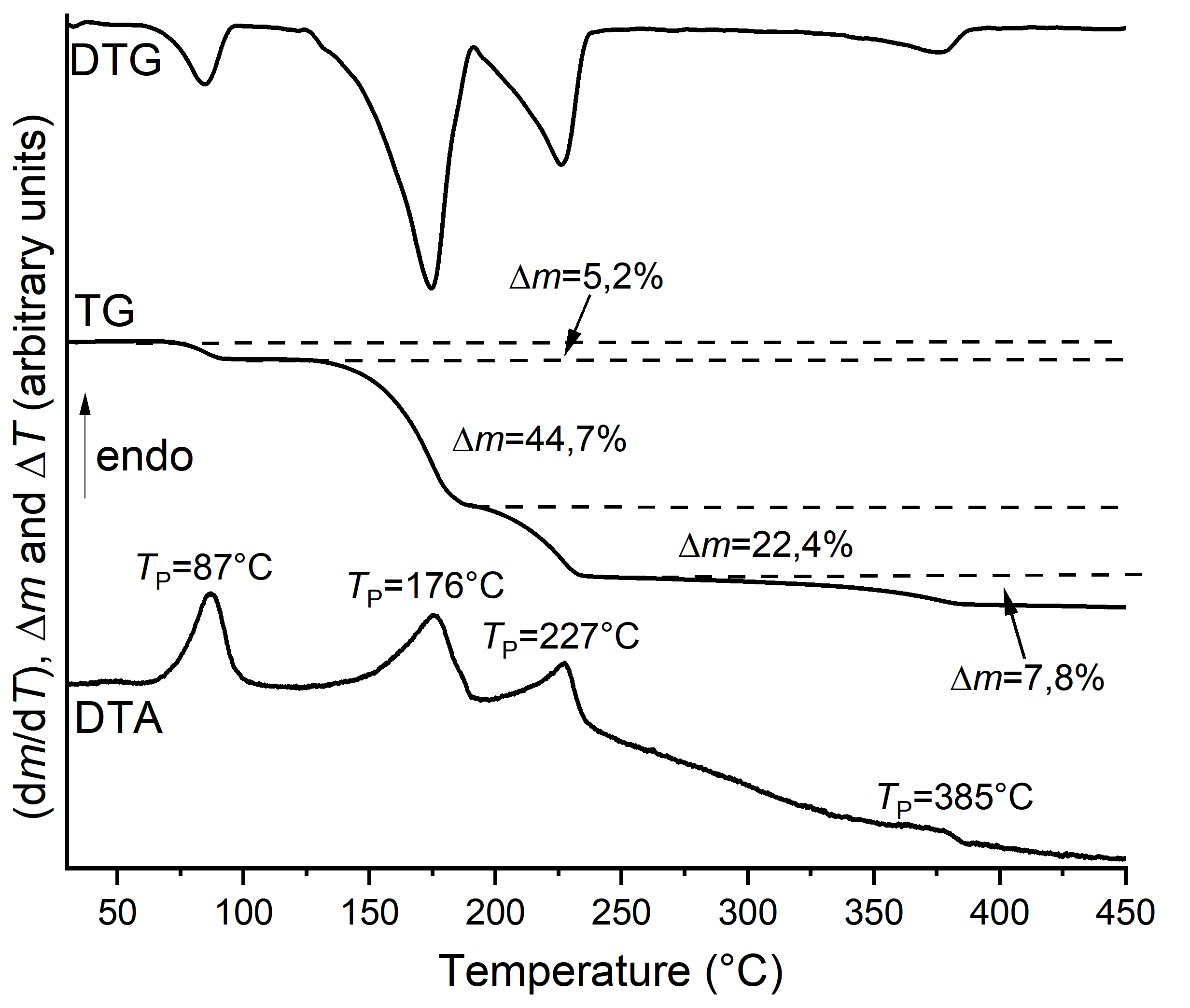

Supplement: Supplementary file 9 [file e-79-01093-sup9.png]

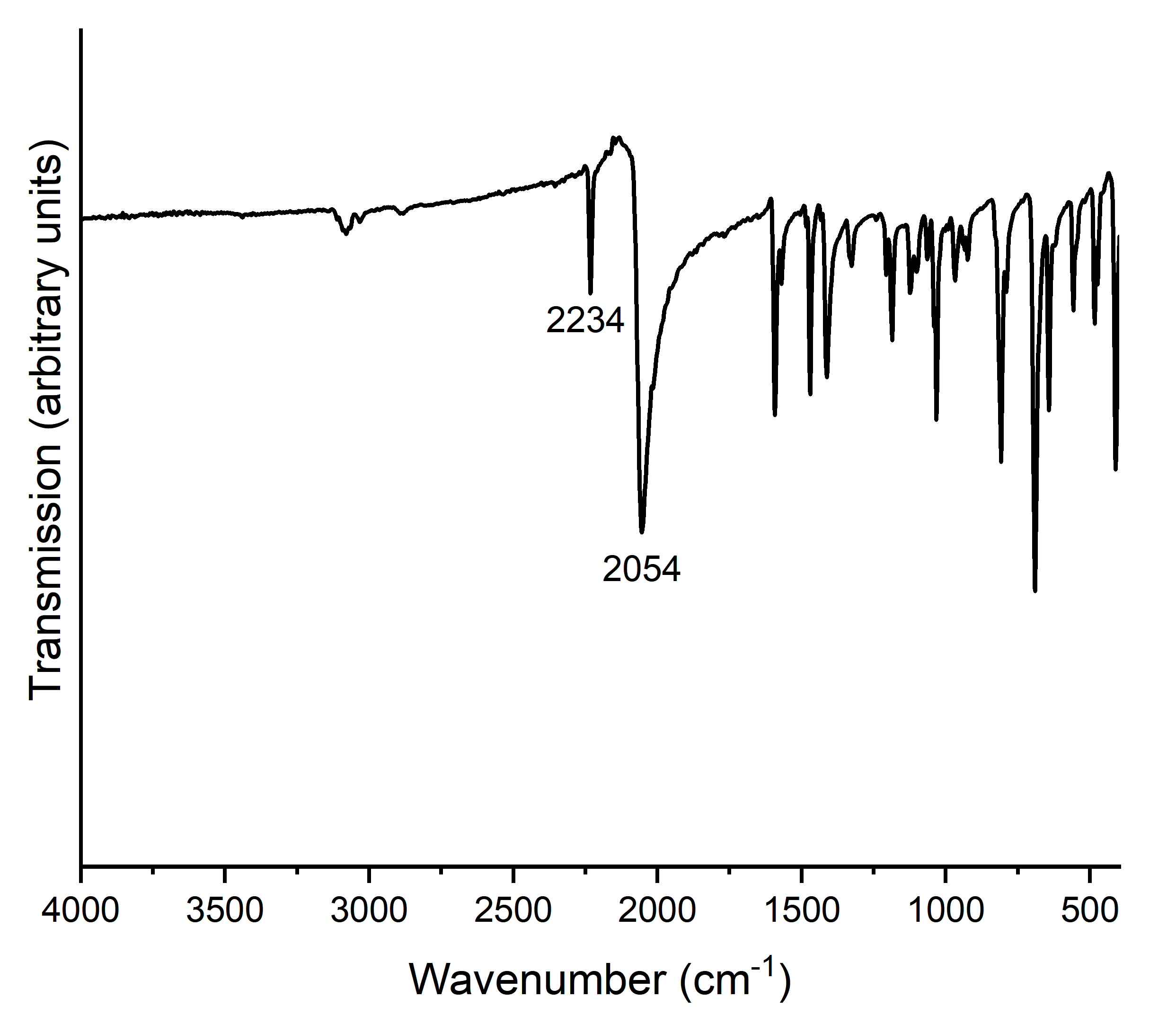

Supplement: Supplementary file 10 [file e-79-01093-sup10.png]

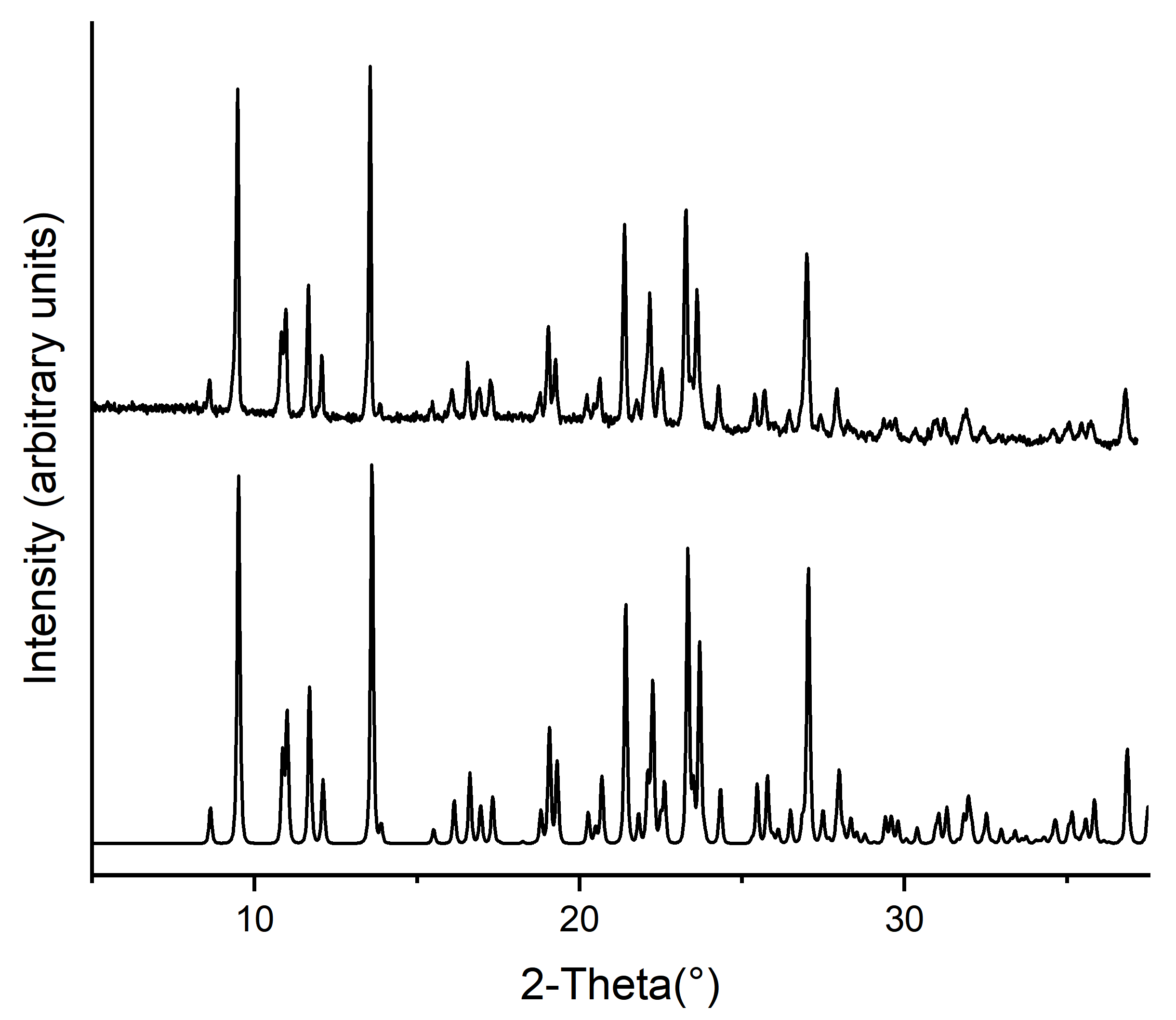

Supplement: Supplementary file 11 [file e-79-01093-sup11.png]

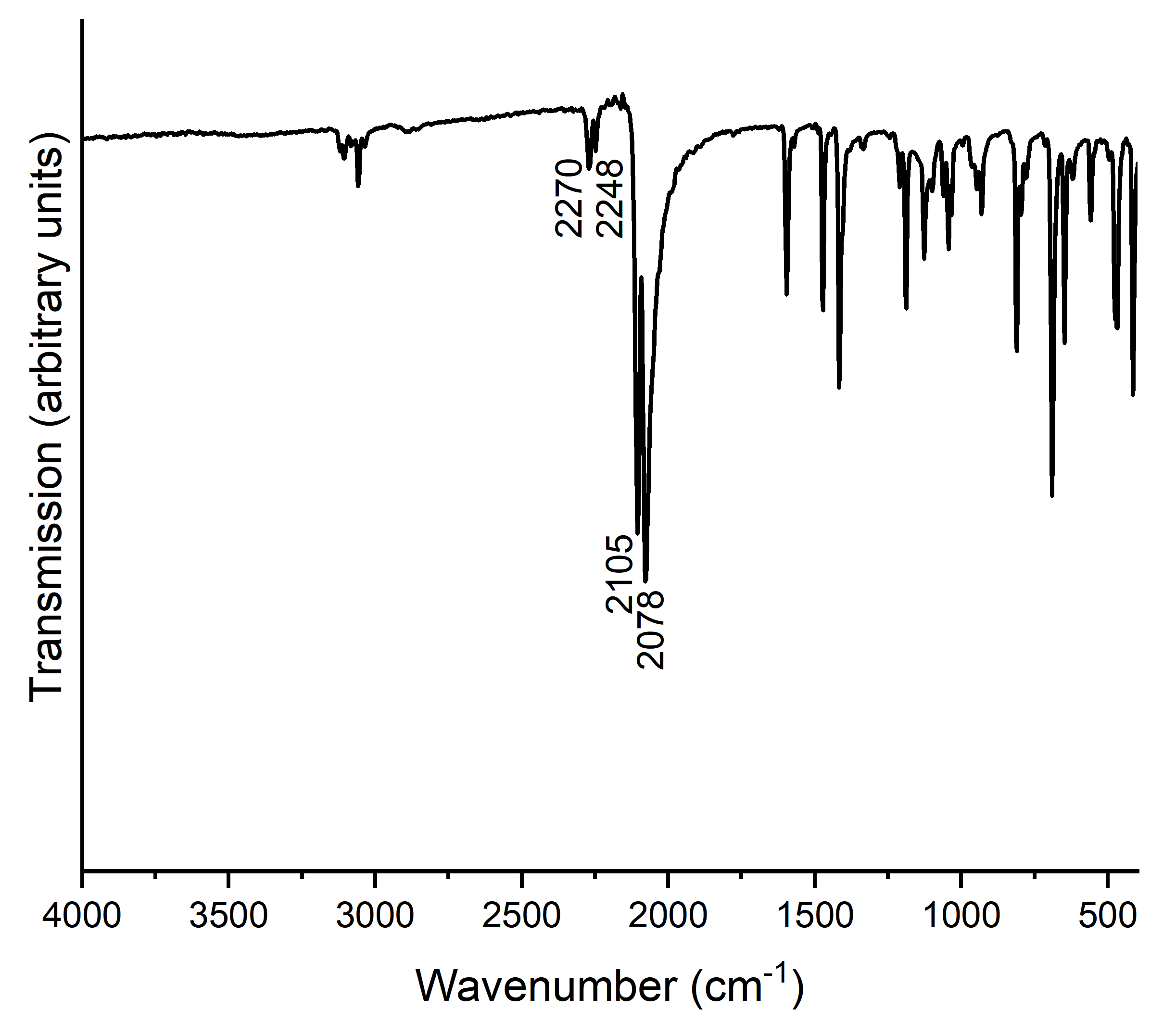

Supplement: Supplementary file 12 [file e-79-01093-sup12.png]

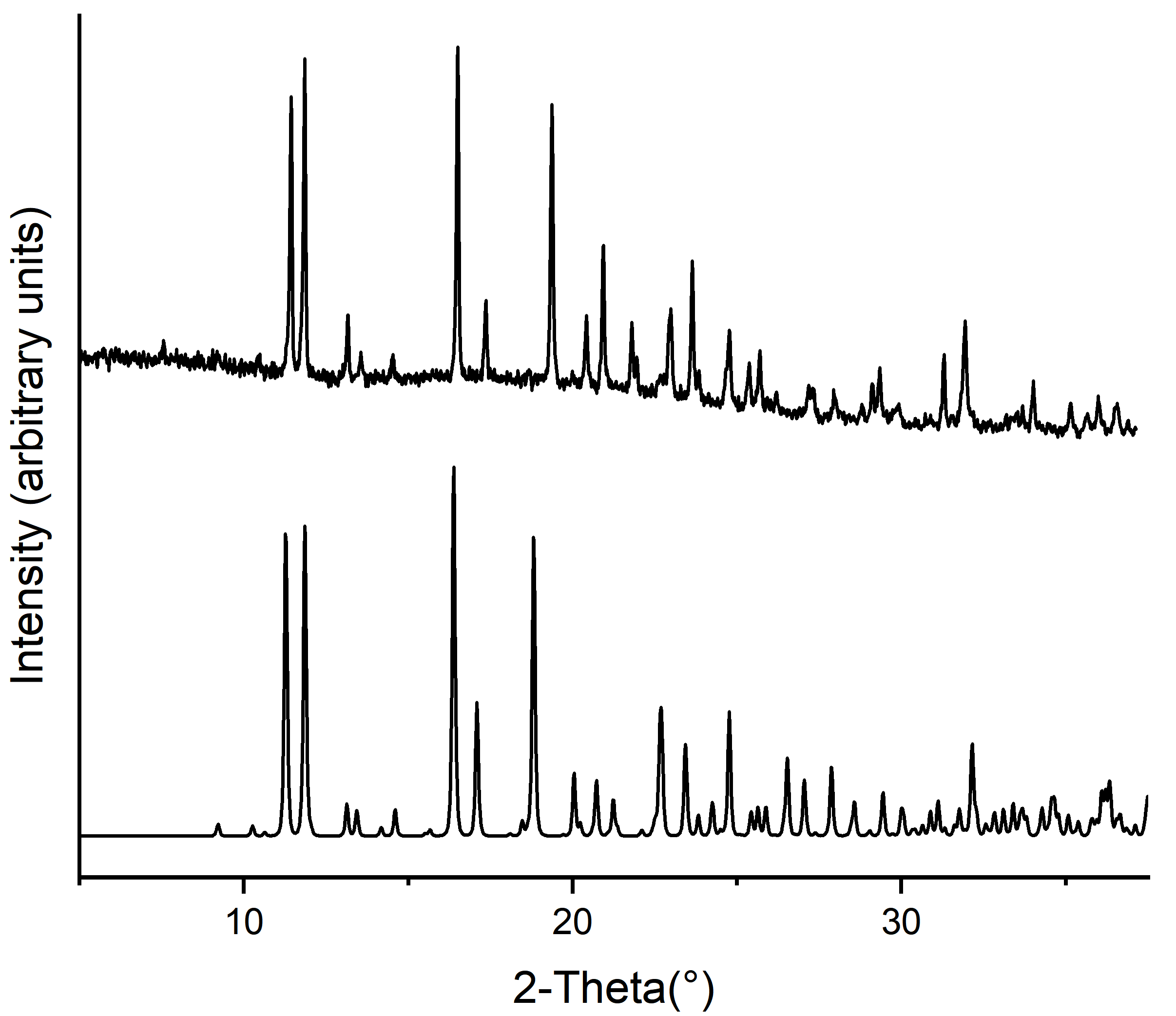

Supplement: Supplementary file 13 [file e-79-01093-sup13.png]
